# Comparison of the Safety and Effectiveness of Apixaban Versus Rivaroxaban in Acute Venous Thromboembolism: A Propensity-Matched Real-World TriNetX Study with Obesity and Cancer Subgroup Analyses

**DOI:** 10.3390/jcm15145410

**Published:** 2026-07-10

**Authors:** Faizan Ahmed, Saifullah Khan, Madeeha Shafqat, Tehmasp Rehman Mirza, Muhammad Abdullah, Najam Gohar, Abdul Hannan, Muhammad Hassan, Muhammad Hussain, Asma Naz, Haris Bin Tahir, Mohammad Saad Saeeduddin, Mohammad Omar Butt, Qaiser Shahzad, Amro Taha, Swapnil Patel, Mohammad Amir Hossain

**Affiliations:** 1 Department of Internal Medicine, Hackensack Meridian Health, Jersey Shore University Medical Center, Neptune, NJ 07753, USA; swapnil.patel@hmhn.org (S.P.); mohammad.hossain@hmhn.org (M.A.H.); 2 Department of Internal Medicine, Dow University of Health Sciences, Karachi 74200, Pakistan; saifrah456@gmail.com (S.K.); abdulhannanoffi@gmail.com (A.H.); azamh5223@gmail.com (M.H.); azamhhh7@gmail.com (M.H.); asmynaz@gmail.com (A.N.); 3 Department of Internal Medicine, Geisinger Medical Center, Danville, PA 17822, USA; drmadeehashafqat@gmail.com; 4 Department of Internal Medicine, Shalamar Medical and Dental College, Lahore 54840, Pakistan; tehmaspmirza@gmail.com (T.R.M.); mohd_abdullah2003@outlook.com (M.A.); 5 Department of Internal Medicine, Ameeruddin Medical College, Post Graduate Medical Institute, Lahore 54000, Pakistan; najamgoharr@gmail.com; 6 Department of Internal Medicine, Lahore General Hospital, Lahore 54000, Pakistan; harristahirchh@gmail.com; 7 Department of Internal Medicine, St. Mary Medical Center, Langhorne, PA 19047, USA; mohammad.saeeduddin@stmaryhealthcare.org; 8 Department of Internal Medicine, Memorial Satilla Health, Waycross, GA 31501, USA; omar9224@gmail.com; 9 Department of Internal Medicine, Winchester Medical Center, Winchester, VA 22601, USA; doctorqaisershahzad@gmail.com; 10 Division of Cardiology, West Virginia University, Morgantown, WV 26506, USA; taha61591@gmail.com

**Keywords:** venous thromboembolism, apixaban, rivaroxaban, direct oral anticoagulants, anticoagulation, major bleeding, TriNetX

## Abstract

**Background:** Apixaban and rivaroxaban are commonly used direct oral anticoagulants for venous thromboembolism (VTE), but comparative real-world effectiveness and safety data remain limited. **Methods:** We conducted a retrospective cohort study using the TriNetX database. Adults with VTE were stratified into apixaban and rivaroxaban treatment groups. Propensity score matching balanced baseline characteristics. Time-to-event outcomes up to 2 years were assessed using Kaplan–Meier analysis and Cox proportional hazards models. **Results:** Following propensity score matching, well-balanced cohorts (8247) were obtained, with a mean age of 58.3 ± 18.0 versus 57.3 ± 18.1 years. Recurrent VTE rates were broadly comparable at 3 months (22.14% vs. 22.26%; HR 1.02, 95% CI 0.95–1.09; *p* = 0.6, ARD −0.001, 95% CI: −0.014 to 0.011), with small but statistically significant increases observed with apixaban at 6-month (HR 1.07, 95% CI 1.01–1.13; *p* = 0.016, ARD 0.009, 95% CI: −0.005 to 0.023), 1-year (HR 1.07, 95% CI 1.01–1.12; *p* = 0.019, ARD 0.004, 95% CI: −0.010 to 0.019), and 2-year follow-ups (HR 1.08, 95% CI 1.04–1.13; *p* = 0.000, ARD −0.001, 95% CI: −0.013 to 0.011). All-cause mortality was comparable through 1 year; however, a statistically significant increase was observed at the 2-year follow-up for apixaban (HR 1.20, 95% CI 1.06–1.37; *p* = 0.005, ARD 0.005, 95% CI: −0.002 to 0.013). Bleeding outcomes were largely comparable in the overall cohort, with no statistically significant differences in composite major bleeding (2-year ARD: −0.004, 95% CI: −0.012–0.004), major bleeding (2-year ARD: 0.002, 95% CI: −0.004 to 0.007), gastrointestinal bleeding (2-year ARD: −0.001, 95% CI: −0.004–0.003), or intracerebral hemorrhage (2-year ARD: 0.000, 95% CI: −0.002–0.003) across follow-up periods. A statistically significant reduction in non-major bleeding with regard to apixaban was observed at the 6-month follow-up only (HR 0.79, 95% CI 0.63–0.98; *p* = 0.033) (2-year ARD: −0.004, 95% CI: −0.010–0.002). Subgroup analyses revealed important heterogeneity: among patients with obesity, composite major bleeding was significantly lower with respect to apixaban at the 2-year follow-up (HR 0.39, 95% CI 0.20–0.76; *p* = 0.004), whereas among patients with cancer, composite major bleeding was significantly higher with apixaban at the 1-year (HR 1.31, 95% CI 1.02–1.70; *p* = 0.037) and 2-year follow-ups (HR 1.32, 95% CI 1.04–1.68; *p* = 0.023). **Conclusions:** In this real-world propensity-matched analysis, apixaban and rivaroxaban demonstrated broadly comparable effectiveness and safety for VTE treatment, with largely similar bleeding outcomes in the overall cohort. Small but statistically significant increases in recurrent VTE and 2-year mortality with apixaban, alongside subgroup-specific bleeding differences, underscore the importance of individualized treatment selection based on patient-specific risk factors.

## 1. Introduction

Venous thromboembolism (VTE) is a significant contributor to cardiovascular disease and associated deaths globally. In developed countries, hundreds to over a million new cases arise annually [1,2]. Involuntary immobility associated with prolonged hospitalization, surgery, and long-term complications due to cancer and an aging population around the world (multiple re-admissions) magnifies the global impact of VTE [3].

Acute VTE without intervention is associated with a high likelihood of progressive pulmonary embolism, additional blood clots, the development of post-thrombotic syndrome, and chronic thromboembolic pulmonary hypertension. This reinforces the continuance of a need for effective, safe, and long-term VTE anticoagulation [4].

The emergence of direct oral anticoagulants (DOACs) in the past few decades has altered the conventional approach to the management of acute VTE and significantly replaced the use of vitamin K antagonists [5]. This has resulted from the predictability of these agents and the reduction in the need for routine monitoring and in medication interactions. The use of DOACs, coupled with the presence of apixaban and rivaroxaban alongside DOACs, has resulted in the consistent prevention of VTE and provision of assurance to patients worldwide [6]. Several observational studies and meta-analyses have compared apixaban and rivaroxaban for the treatment of VTE. Overall, both have comparable effectiveness in preventing recurrent VTE, whereas some studies have suggested there is a lower risk of major bleeding with apixaban. However, the findings are not uniform across different patient demographics and follow-up durations. However, despite significant application, the available studies on the comparison of these agents in the real world in high-risk patient populations, in the presence of obesity and/or aggravating cancer, have remained within the scope of limited temporal and objective measurement.

Both obesity and cancer are important factors in describing how VTE risk and response to anticoagulants are balanced. Both conditions can affect how drugs are distributed in the body and the risk of side effects [7,8,9]. Neither obesity, cancer, nor chemotherapy permits reliable anticoagulant pharmacokinetics, frequently disrupting the delicate balance between thrombotic and bleeding risks. Guidelines offer limited specific direction, often defaulting to low-molecular-weight heparin (LMWH) or select DOACs amid uncertainty [10,11].

Thus, using the TriNetX global database, we implemented an extensive safety-and-effectiveness trial comparing the outcomes of apixaban and rivaroxaban in the real world. We specifically focused on the outcomes of recurrences of venous thromboembolism, major bleeding, and overall mortality and conducted subgroup analyses for the high-risk phenotypes of obesity and cancer that affect the treatment outcomes. The aim of the trial was to use real-world data to analyze treatment effects and risk–benefit beyond the scope of conventional randomized clinical trials.

## 2. Methods

### 2.1. Data Source

This observational study was conducted using the TriNetX Research network, a global federated platform providing access to de-identified electronic medical records (EMRs). The data analysis was conducted on 19 April 2026, drawing from a diverse pool of 169 healthcare organizations (HCOs) within the Global Collaborative Network. TriNetX aggregates patient-level clinical data, including medications, diagnoses, and lab results, in accordance with the Health Insurance Portability and Accountability Act (HIPAA). As this study utilized fully de-identified data from the TriNetX Research Network, no identifiable human subject data were accessed. In accordance with institutional policies governing secondary use of de-identified data, formal institutional review board (IRB) approval was not required, and the study was considered exempt/non-human-subject research. The study was conducted and reported in accordance with the Strengthening the Reporting of Observational Studies in Epidemiology (STROBE) guidelines [12].

### 2.2. Study Population and Design

The study included patients aged 18 years or older diagnosed with acute venous thromboembolism (VTE), which encompassed pulmonary embolism and acute embolism or thrombosis of deep veins in the lower or upper extremities. To ensure clinical relevance, the diagnosis was required to be associated with an emergency, inpatient acute, or inpatient non-acute hospital visit. Individuals were excluded if they had a history of prosthetic heart valves or hepatic failure or were pregnant. For the primary base analysis, patients with malignant neoplasms were also excluded.

Patients were categorized into two cohorts based on their anticoagulant treatment: the Apixaban cohort (Cohort A) and the Rivaroxaban cohort (Cohort B) (Supplementary Tables S1 and S2). All medications were identified using RxNorm Concept Unique Identifiers (RXCUIs) within the TriNetX database. Apixaban was defined using RXCUI 1364430, and rivaroxaban was identified using RXCUI 1114195. The full list of medication codes used to define exposure is provided in the Supplementary Materials for transparency and reproducibility. This approach ensured consistent identification of direct oral anticoagulant exposure across all participating healthcare organizations. Exposure was defined as documented prescription or administration of apixaban or rivaroxaban occurring within the same encounter or within 7 days of the index VTE diagnosis. Patients were assigned according to the first direct oral anticoagulant prescribed. The index event was defined as the date a patient first satisfied both the diagnostic criteria for VTE and the initiation of the respective medication. This temporal alignment between VTE diagnosis and anticoagulant initiation was used to minimize immortal time bias. Analyses were conducted using an intention-to-treat approach, whereby patients remained assigned to their initial anticoagulant cohort throughout follow-up. Detailed information regarding loading versus maintenance dosing, dose adjustments, medication adherence, treatment persistence, temporary interruptions, treatment discontinuation, switching between anticoagulants, and crossover during follow-up was not reliably available within the TriNetX platform and therefore could not be incorporated into the analysis.

Patients were followed from the index date until outcome occurrence, death, loss to follow-up, or completion of the predefined follow-up interval, whichever occurred first. Censoring at the end of the available observation period was applied when it was outcome-free. Subgroup analyses were also performed for patients with active cancer, defined as any malignancy diagnosis (ICD-10 codes: C00–C99) recorded within 12 months prior to the index date and those with a body weight exceeding 120 kg (265 lbs), a threshold selected to correspond with the exclusion criterion used in the COBRRA randomized trial and consistent with NICE guidance identifying an absolute body weight > 120 kg as a threshold for heightened concern regarding DOAC pharmacokinetic adequacy [13,14]. The obesity subgroup was defined using recorded vital sign weight data in TriNetX, not ICD-10 BMI diagnosis codes; BMI-range codes appearing in the baseline characteristics table were included descriptively only.

### 2.3. Study Endpoints

The analysis evaluated clinical outcomes over follow-up periods of 3 months, 6 months, 1 year, and 2 years. The primary outcome was Recurrent VTE, defined as a composite of pulmonary embolism and deep-vein thrombosis. Recurrent VTE events were identified using ICD-10 diagnostic codes (I26, I82.4, and I82.6) recorded more than 30 days after the index date; this 30-day lag period was applied to exclude persistent or follow-up coding of the index episode, repeat encounter codes during acute management, and chronic or carry-forward VTE codes that might have appeared in the immediate post-index period. Only VTE diagnoses recorded after this washout window were captured as recurrent events. Secondary outcomes include all-cause mortality, composite major bleeding, major bleeding, non-major bleeding, gastrointestinal bleeding and intracerebral hemorrhage (ICH). All bleeding outcomes were identified exclusively through ICD-10 and CPT diagnosis codes recorded in the TriNetX research network. No adjudication based on laboratory parameters, transfusion requirements, or procedural intervention was performed, consistent with the administrative-claim-based nature of the dataset. Bleeding outcomes were defined as follows: composite major bleeding (I60, I61, I62, K25.0, K25.4, K26.4, K26.0, M25.0, H35.6, K92.2, D62, R58, R31, N93.9 and CPT 36340), major bleeding (I60, I61, I62, K25.0, K25.4, K26.0, K26.4, M25.0, H35.6, D62, and K92.2), non-major bleeding (R58, N93.9, and R31), GI bleeding (K92.2, K25.0, K25.4, K26.0, and K26.4), and ICH (I60, I61, and I62).

### 2.4. Statistical Analysis

To mitigate baseline differences between the cohorts, propensity score matching (PSM) was conducted. For the primary 1-year follow-up analysis, Cohort A (*n* = 22,629) and Cohort B (*n* = 9449) were matched in a 1:1 ratio, resulting in balanced groups of 9308 patients each [15]. Matching criteria included demographic factors (age, sex, and race) as well as various comorbid conditions and prior medications. Nearest-neighbor propensity score matching without replacement was performed within the TriNetX platform using a caliper width of 0.1 pooled standard deviations. Covariate balance after matching was assessed using standardized mean differences (SMDs), with values < 0.1 considered indicative of an acceptable balance.

Incidence risks and measures of association, specifically Risk Ratios (RRs) and Odds Ratios (ORs) with 95% confidence intervals (CIs), were calculated through the TriNetX Compare Outcomes model. Time-to-event data were visualized using Kaplan–Meier survival curves, and differences between cohorts were tested with the log-rank test. Hazard ratios (HRs) and 95% CIs were derived from Cox proportional hazards regression models employed within the TriNetX platform [16,17]. HRs were considered the primary time-to-event effect measure. Proportional hazards assumptions were not directly testable within the TriNetX interface. A two-sided *p*-value of less than 0.05 was used to determine statistical significance for all tests.

## 3. Results

### 3.1. Patient Characteristics

**Figure 1:**
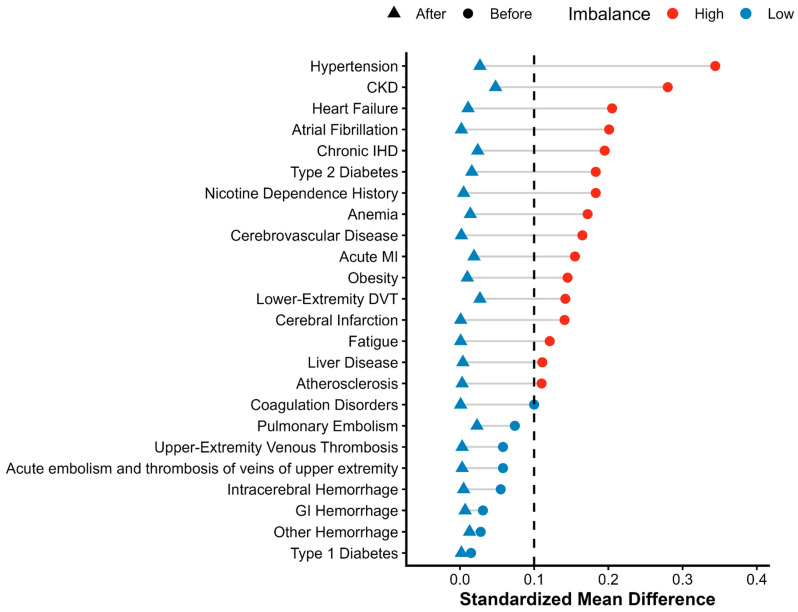
Love plot presenting standardized mean differences (SMDs) for baseline comorbidities before and after propensity score matching.

**Figure 2:**
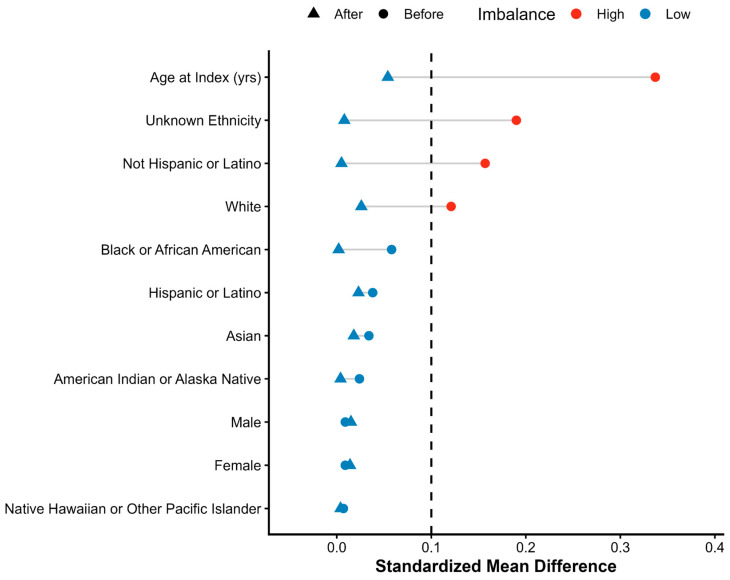
Love plot presenting standardized mean differences (SMDs) for baseline demographic characteristics before and after propensity score matching.

**Figure 3:**
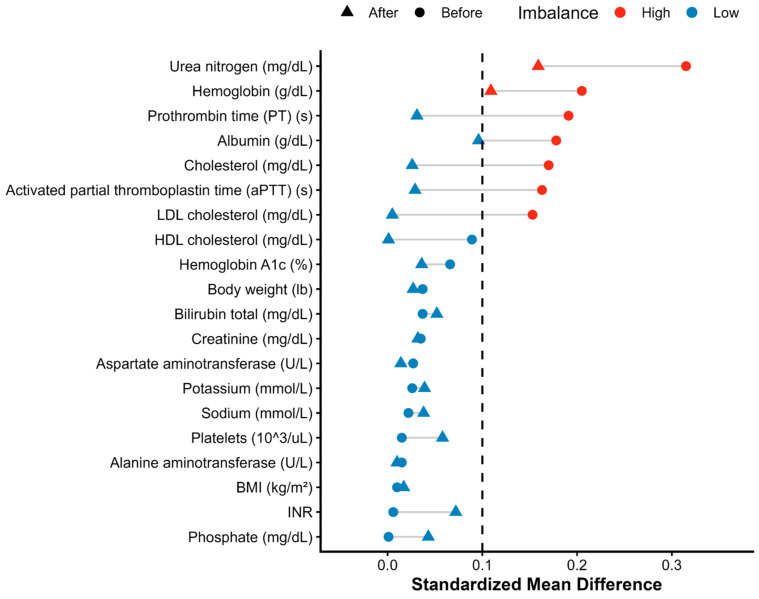
Love plot presenting standardized mean differences (SMDs) for baseline laboratory parameters before and after propensity score matching.

**Figure 4:**
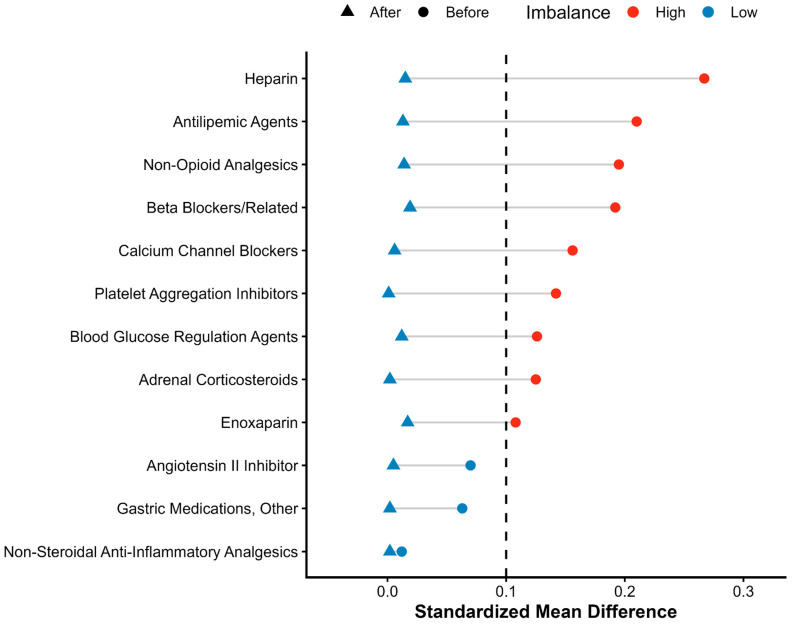
Love plot presenting standardized mean differences (SMDs) for baseline drug use before and after propensity score matching.

A total of 35,589 patients were identified, including 26,796 in the apixaban and VTE group and 8793 in the rivaroxaban and VTE group, before propensity score matching (PSM) (Figure 1, Figure 2, Figure 3 and Figure 4). After matching, there were 8247 in each group (Supplementary Figure S1).

Before propensity score matching, patients in the apixaban group were older than those in the rivaroxaban group (62.7 ± 16.9 vs. 56.8 ± 18.1 years; SMD, 0.337), while the sex distribution was similar (males: 54.10% vs. 54.50%). The apixaban cohort demonstrated a higher prevalence of comorbidities, including hypertensive disease (41.20% vs. 25.20%; SMD, 0.344), heart failure (11.90% vs. 6.00%; SMD, 0.205), chronic kidney disease (9.60% vs. 2.90%; SMD, 0.28), and type 2 diabetes mellitus (15.60% vs. 9.60%; SMD, 0.183). Concomitant medication use was also more frequent among apixaban users, particularly platelet aggregation inhibitors (18.70% vs. 13.50%) and heparin (40.80% vs. 28.20%). Laboratory findings were broadly comparable, although hemoglobin levels were slightly lower (12.1 ± 2.4 vs. 12.5 ± 2.3 g/dL) and blood urea nitrogen levels were slightly higher (18.7 ± 12.2 vs. 15.4 ± 8.1 mg/dL) in the apixaban cohort (Table 1) (Figure 1, Figure 2, Figure 3 and Figure 4).

Following propensity score matching, baseline characteristics became well-balanced between the cohorts. The mean ages were identical (58.3 ± 18.0 vs. 57.3 ± 18.1 years; SMD, 0.054), and key comorbidities such as hypertensive disease (27.50% vs. 26.30%), heart failure (6.50% vs. 6.30%), chronic kidney disease (4.00% vs. 3.10%), and type 2 diabetes mellitus (10.50% vs. 10.00%) were closely aligned. Medication use and laboratory parameters also became comparable, with nearly all SMD values reduced to below 0.1, indicating successful matching and balanced cohorts suitable for comparative outcome analysis (Table 1) (Figure 1, Figure 2, Figure 3 and Figure 4).

Overall, propensity score matching effectively minimized baseline imbalances, resulting in two well-balanced cohorts suitable for reliable comparative outcome analysis.

### 3.2. Primary Outcomes

#### 3.2.1. Recurrent Venous Thromboembolism (VTE)

**Figure 5:**
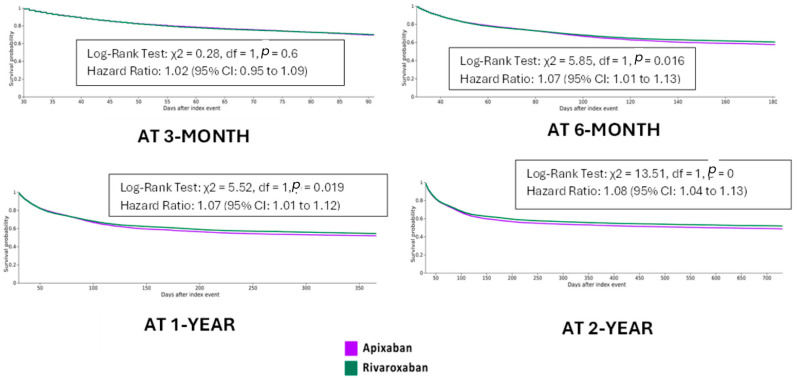
Recurrent VTE outcomes in apixaban and rivaroxaban groups at all follow-ups.

**Figure 6:**
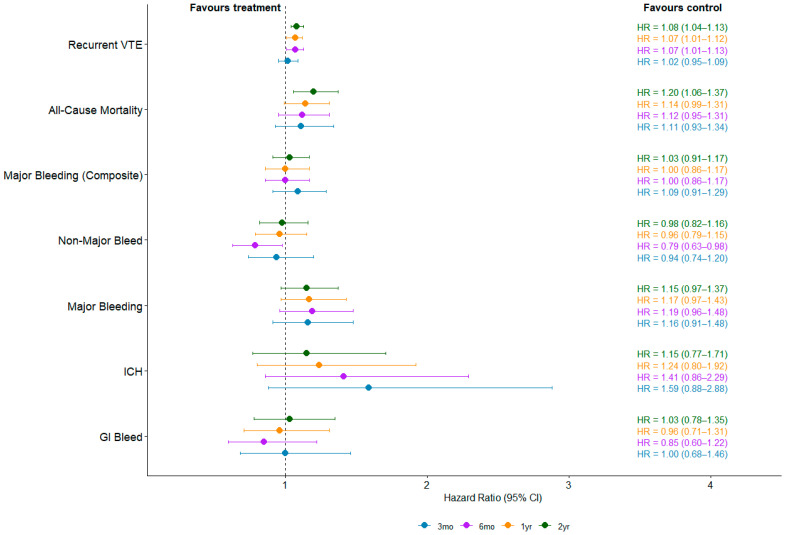
Forest plot of primary clinical outcomes comparing apixaban versus rivaroxaban in the overall propensity-matched acute venous thromboembolism cohort.

At the 3-month follow-ups, 22.14% and 22.26% recurrences of VTE were observed in patients in the apixaban and rivaroxaban groups, respectively. Kaplan–Meier analysis yielded a hazard ratio (HR) of 1.02 (95% CI: 0.95–1.09; log-rank *p* = 0.6). At the 6-month follow-up, the absolute rates were 29.79% vs. 28.87%, respectively, and analysis yielded an HR of 1.07 (95% CI: 1.01–1.13; log-rank *p* = 0.016). At the 1-year follow-up, the recurrence rates were 32.92% vs. 32.48%, respectively, with analysis yielding an HR of 1.07 (95% CI: 1.01–1.12; log-rank *p* = 0.019). At the 2-year follow-up, the rates were 35.75% vs. 35.83%, respectively, and analysis demonstrated an HR of 1.08 (95% CI: 1.04–1.13; log-rank *p* = 0.000). Overall, a slight difference among groups was noted. Despite statistical significance at 6 months, 1 year, and 2 years, these findings should be interpreted cautiously, given the modest absolute differences and potential influence of sample size. The absolute risk difference (ARD) at 3 months was −0.001 (95% CI: −0.014 to 0.011), while that at 6 months was 0.009 (95% CI: −0.005 to 0.023), that at 1 year was 0.004 (95% CI: −0.010 to 0.019), and that at 2 years was −0.001 (95% CI: −0.013 to 0.011) (Figure 5 and Figure 6) (Table 2).

**Figure 7:**
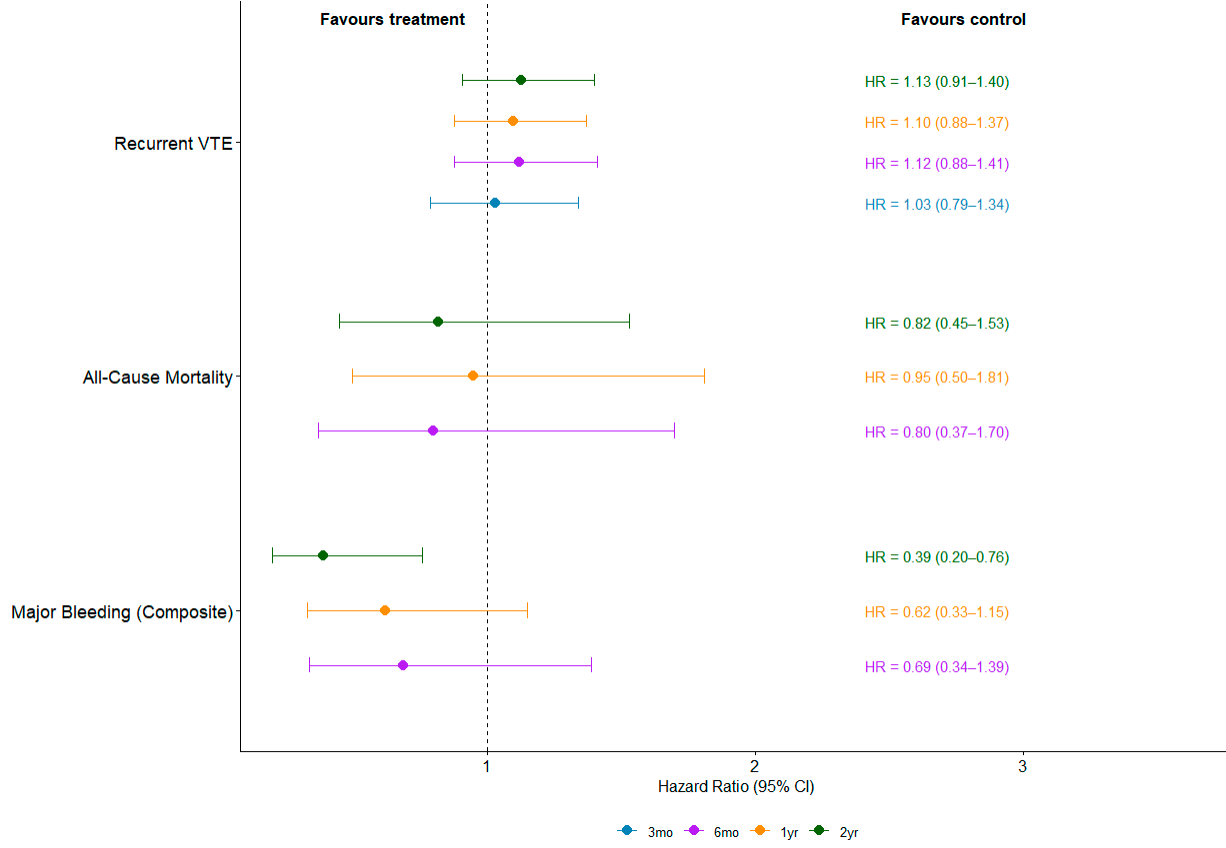
Forest plot of clinical outcomes comparing apixaban versus rivaroxaban in patients with obesity and acute venous thromboembolism.

**Figure 8:**
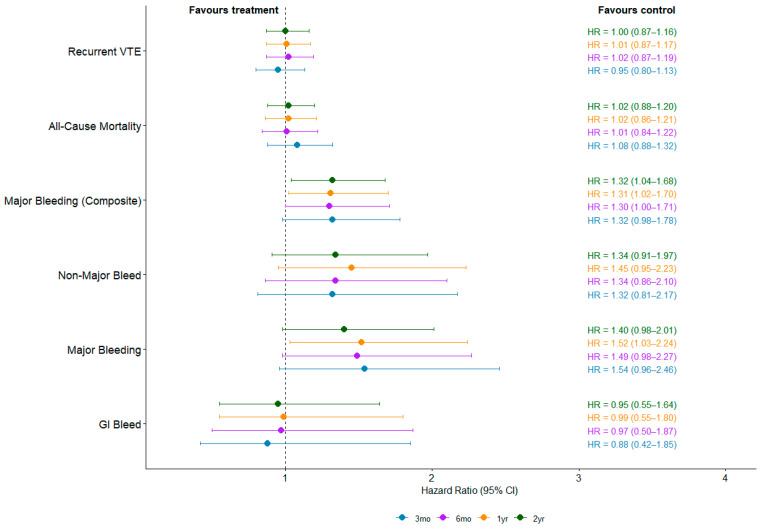
Forest plot of clinical outcomes comparing apixaban versus rivaroxaban in patients with cancer and acute venous thromboembolism.

The results of the subgroup analysis for the obese population at 3 months were 22.93% and 21.90% [HR: 1.03 (95% CI: 0.79–1.34); log-rank *p* = 0.837], at 6 months were 31.20% and 27.89% [HR: 1.12 (95% CI: 0.88–1.41); log-rank *p* = 0.355], at 1 year were 34.30% and 31.61% [HR: 1.10 (95% CI: 0.88–1.37); log-rank *p* = 0.394], and at 2 years were 36.36% and 33.06% [HR: 1.13 (95% CI: 0.91–1.40); log-rank *p* = 0.263] for the apixaban and rivaroxaban groups, respectively, indicating statistically non-significant differences in recurrence (Figure 7 and Supplementary Figure S2) (Supplementary Table S5). The results of the subgroup analysis by cancer at 3 months were 22.12% and 24.09% [HR: 0.95 (95% CI: 0.80–1.13); log-rank *p* = 0.57], at 6 months were 29.15% and 29.71% [HR: 1.02 (95% CI: 0.87–1.19); log-rank *p* = 0.832], at 1 year were 31.40% and 32.52% [HR: 1.01 (95% CI: 0.87–1.17); log-rank *p* = 0.897], and at 2 years were 32.80% and 34.40% [HR: 1.00 (95% CI: 0.87–1.16); log-rank *p* = 0.959] for the apixaban and rivaroxaban groups, respectively. These results show a non-significant difference in recurrence between both groups (Figure 8 and Supplementary Figure S3) (Supplementary Table S6).

#### 3.2.2. Sensitivity Analysis for Recurrent VTE

**Figure 9:**
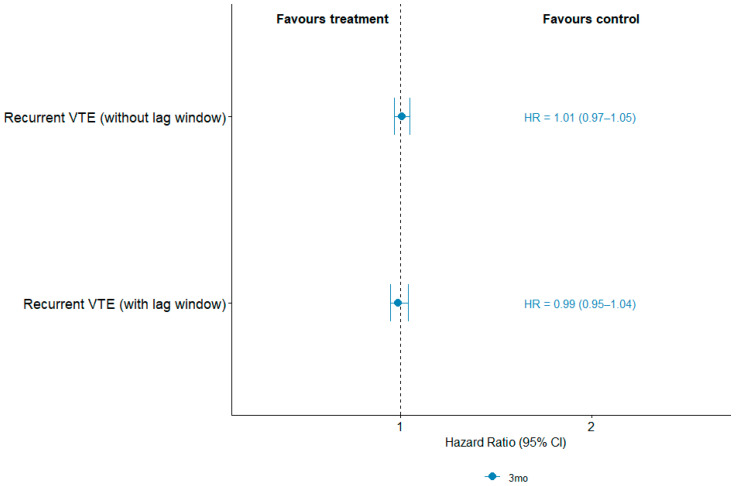
Forest plot of sensitivity analysis of recurrent VTE comparing apixaban versus rivaroxaban in the overall propensity-matched acute venous thromboembolism cohort.

The recurrence rates of VTE at 3 months were 49.61% and 49.59% in the apixaban and rivaroxaban groups, with an HR of 1.01 (95% CI: 0.97–1.05; log-rank *p* = 0.682). Due to these high recurrence rates, a separate analysis from day 1 to day 30 was run, which yielded rates of 42.83% and 43.30% for the apixaban and rivaroxaban groups, with an HR of 0.99 (95% CI: 0.95–1.04; log-rank *p* = 0.771). The high event rates in the initial month suggest that the primary VTE event might have been coded as recurrent VTE during acute-phase hospitalization in the database. Eliminating this artifact limited the inflated recurrence rates for both groups. Nonetheless, the similar effect estimates observed in the sensitivity analysis support the robustness of the comparative findings and are consistent with the overall neutral treatment effect reported in the current literature (Figure 9) (Supplementary Table S7).

### 3.3. Secondary Outcomes

#### 3.3.1. All-Cause Mortality

At the 3-month follow-up, death rates of 2.95% and 2.70% were recorded among patients administered apixaban and rivaroxaban, respectively. Analysis yielded an HR of 1.11 (95% CI: 0.93–1.34; log-rank *p* = 0.247). At the 6-month follow-up, (apixaban vs. rivaroxaban: 3.92% vs. 3.60%) analysis yielded an HR of 1.12 (95% CI: 0.95–1.31; log-rank *p* = 0.171). At the 1-year follow-up, mortality with an HR of 1.14 (95% CI: 0.99–1.31; log-rank *p* = 0.064) was observed. At the 2-year follow-up, an HR of 1.20 (95% CI: 1.06–1.37; log-rank *p* = 0.005) was calculated. HR estimates were greater than 1.0 at all follow-up time points, reaching statistical significance at the 2-year follow-up. The ARDs at follow-up were 0.003 (95% CI: −0.003 to 0.008) for 3 months, 0.003 (95% CI: −0.003 to 0.009) for 6 months, 0.004 (95% CI: −0.003 to 0.010) for 1 year, and 0.005 (95% CI: −0.002 to 0.013) for 2 years (Figure 6 and Supplementary Figure S4) (Table 2).

Mortality in the obesity subgroup at 6 months was 2.49% and 3.11% [HR: 0.80 (95% CI: 0.37–1.70); log-rank *p* = 0.555]. A follow-up at 1 year yielded an HR of 0.95 (95% CI: 0.50–1.81; log-rank *p* = 0.871), and one at 2 years yielded an HR of 0.82 (95% CI: 0.45–1.53; log-rank *p* = 0.538). This translates to comparable, albeit non-significant, mortality in both groups (Figure 7 and Supplementary Figure S5) (Supplementary Table S5). Mortality in the cancer subgroup at 3 months was 17.58% and 16.62% [HR: 1.08 (95% CI: 0.88–1.32); log-rank *p* = 0.483] and that at 6 months was 21.55% and 21.81% [HR: 1.01 (95% CI: 0.84–1.22); log-rank *p* = 0.889] for the apixaban and rivaroxaban groups, respectively. A follow-up at 1 year resulted in an HR of 1.02 (95% CI: 0.86–1.21; log-rank *p* = 0.816), and one at 2 years yielded an HR of 1.02 (95% CI: 0.88–1.20; log-rank *p* = 0.768). Thus, in the cancer subgroup, a non-significant difference in mortality was recorded (Figure 8 and Supplementary Figure S6) (Supplementary Table S6).

#### 3.3.2. Major Bleeding (Composite)

At the 3-month follow-up, the rates of major bleeding (composite) were 3.61% and 3.38% in patients receiving apixaban and rivaroxaban, respectively. Analysis yielded an HR of 1.09 (95% CI: 0.91–1.29; log-rank *p* = 0.354). At the 6-month follow-up, analysis yielded an HR of 1.00 (95% CI: 0.86–1.17; log-rank *p* = 0.99). Similarly, at the 1-year follow-up, analysis yielded an HR of 1.00 (95% CI: 0.86–1.17; log-rank *p* = 0.62). At the 2-year follow-up, analysis resulted in an HR of 1.03 (95% CI: 0.91–1.17; log-rank *p* = 0.641). This shows comparable, albeit non-significant, instances of bleeding in both groups. ARDs were 0.002 (95% CI: −0.004 to 0.008) at 3 months, −0.001 (95% CI: −0.007 to 0.006) at 6 months, −0.001 (95% CI: −0.008 to 0.006) at 1 year and −0.004 (95% CI: −0.012 to 0.004) at 2 years (Figure 6 and Supplementary Figure S7) (Table 2).

Subgroup analysis for the obese population at 6 months yielded 2.95% and 4.32% [HR: 0.69 (95% CI: 0.34–1.39); log-rank *p* = 0.296] for the apixaban and rivaroxaban groups, respectively. A follow-up at 1 year yielded an HR of 0.62 (95% CI: 0.33–1.15; log-rank *p* = 0.127), and one at 2 years yield an HR of 0.39 (95% CI: 0.20–0.76; log-rank *p* = 0.004). The differences are non-significant, except for the 2-year follow-up (Figure 7 and Supplementary Figure S8) (Supplementary Table S5). Subgroup analysis by cancer at 3 months yielded 11.46% and 8.88% [HR: 1.32 (95% CI: 0.98–1.78); log-rank *p* = 0.065]. The HR at 6 months was 1.30 (95% CI: 1.00–1.71; log-rank *p* = 0.054), that at 1 year was 1.31 (95% CI: 1.02–1.70; log-rank *p* = 0.037), and that at 2 years was 1.32 (95% CI: 1.04–1.68; log-rank *p* = 0.023). Thus, in contrast to the obese subgroup, the cancer subgroup had an HR consistently greater than 1, which was significant for the 1-year and 2-year follow-ups (Figure 8 and Supplementary Figure S9) (Supplementary Table S6).

#### 3.3.3. Non-Major Bleeding

At the 3-month follow-up, occurrences of 1.59% and 1.71% were recorded in the apixaban group and rivaroxaban group, respectively. Kaplan–Meier analysis yielded an HR of 0.94 (95% CI: 0.74–1.20; log-rank *p* = 0.635). At the 6-month follow-up, analysis yielded an HR of 0.79 (95% CI: 0.63–0.98; log-rank *p* = 0.033) for non-major bleeding. For the follow-up at 1 year, analysis yielded an HR of 0.96 (95% CI: 0.79–1.15; log-rank *p* = 0.634), and at the 2-year follow-up, analysis resulted in an HR of 0.98 (95% CI: 0.82–1.16; log-rank *p* = 0.78). This demonstrates a comparable, non-significant bleeding risk among both groups, except for the 6-month follow-up. The ARDs calculated were −0.001 (95% CI: −0.005 to 0.003) at 3 months, −0.005 (95% CI: −0.010 to −0.001) at 6 months, −0.003 (95% CI: −0.008 to 0.002) at 1 year and −0.004 (95% CI: −0.010 to 0.002) at 2 years (Figure 6 and Supplementary Figure S10) (Table 2).

Subgroup analysis by cancer at 3 months yielded 3.66% and 2.82% [HR: 1.32 (95% CI: 0.81–2.17); log-rank *p* = 0.264] for the apixaban and rivaroxaban groups, respectively. The HR at 6 months was 1.34 (95% CI: 0.86–2.10; log-rank *p* = 0.197), that at 1 year was 1.45 (95% CI: 0.95–2.23; log-rank *p* = 0.085), and that at 2 years was 1.34 (95% CI: 0.91–1.97; log-rank *p* = 0.142). The HRs were higher than 1; however, the risk was not statistically significant (Supplementary Figure S11) (Figure 8 and Supplementary Table S6).

#### 3.3.4. Major Bleeding

At the 3-month follow-up, major bleeding rates of 1.76% and 1.55% were observed among patients administered apixaban and rivaroxaban, respectively. Analysis yielded an HR of 1.16 (95% CI: 0.91–1.48; log-rank *p* = 0.24). At the 6-month follow-up, analysis resulted in an HR of 1.19 (95% CI: 0.96–1.48; log-rank *p* = 0.12). At the 1-year follow-up, analysis showed an HR of 1.17 (95% CI: 0.97–1.43; log-rank *p* = 0.109). At the 2-year follow-up, analysis yielded an HR of 1.15 (95% CI: 0.97–1.37; log-rank *p* = 0.113). This demonstrates that the differences across groups for major bleeding were not statistically significant. At the follow-ups, the ARD for 3 months was 0.002 (95% CI: −0.002 to 0.006), that for 6 months was 0.003 (95% CI: −0.001 to 0.008), that for 1 year was 0.003 (95% CI: −0.002 to 0.008), and that for 2 years was 0.002 (95% CI: −0.004 to 0.007) (Supplementary Figure S12) (Table 2).

Subgroup analysis by cancer at 3 months yielded 4.64% and 3.05% [HR: 1.54 (95% CI: 0.96–2.46); log-rank *p* = 0.068] for the apixaban and rivaroxaban groups, respectively. The HR at 6 months was 1.49 (95% CI: 0.98–2.27; log-rank *p* = 0.059), that at 1 year was 1.52 (95% CI: 1.03–2.24; log-rank *p* = 0.033), and that at 2 years was 1.40 (95% CI: 0.98–2.01; log-rank *p* = 0.062). Except for the 1-year follow-up, the differences across groups were non-significant (Figure 8 and Supplementary Figure S13) (Supplementary Table S6).

#### 3.3.5. Intracerebral Hemorrhage (ICH)

At the 3-month follow-up, ICH was recorded in 0.34% and 0.22% of patients receiving apixaban and rivaroxaban, respectively. Analysis yielded an HR of 1.59 (95% CI: 0.88–2.88; log-rank *p* = 0.119). At the 6-month follow-up, the HR was 1.41 (95% CI: 0.86–2.29; log-rank *p* = 0.168). At the 1-year follow-up, analysis yielded an HR of 1.24 (95% CI: 0.80–1.92; log-rank *p* = 0.345), and at the 2-year follow-up, an HR of 1.15 (95% CI: 0.77–1.71; log-rank *p* = 0.487) was obtained. These results indicate a statistically non-significant difference, with an HR higher than 1; however, a declining trend in the risk magnitude was also observed. The ARDs calculated were 0.001 (95% CI: 0.000 to 0.003) at 3 months, 0.001 (95% CI: −0.001 to 0.003) at 6 months, 0.001 (95% CI: −0.001 to 0.003) at 1 year, and 0.000 (95% CI: −0.002 to 0.003) at 2 years (Figure 6 and Supplementary Figure S14) (Table 2).

#### 3.3.6. Gastrointestinal (GI) Bleeding

At the 3-month follow-up, GI bleeding rates of 0.64% and 0.65% were recorded in patients administered apixaban and rivaroxaban, respectively. Analysis yielded an HR of 1.00 (95% CI: 0.68–1.46; log-rank *p* = 0.988). At the 6-month follow-up, analysis yielded an HR of 0.85 (95% CI: 0.60–1.22; log-rank *p* = 0.385). At the 1-year follow-up, the HR was 0.96 (95% CI: 0.71–1.31; log-rank *p* = 0.812), and at the 2-year follow-up, the HR was 1.03 (95% CI: 0.78–1.35; log-rank *p* = 0.834). This demonstrates a comparable risk of GI bleeding in both groups that was statistically non-significant. At the follow-ups, the ARDs were 0.000 (95% CI: −0.003 to 0.002) for 3 months, −0.001 (95% CI: −0.004 to 0.001) for 6 months, −0.001 (95% CI: −0.004 to 0.002) for 1 year, and −0.001 (95% CI: −0.004 to 0.003) for 2 years (Figure 6 and Supplementary Figure S15) (Table 2).

In subgroup analysis by cancer at 3 months, the GI bleeding rates were 1.26% and 1.45% [HR: 0.88 (95% CI: 0.42–1.85); log-rank *p* = 0.733] in the apixaban and rivaroxaban groups, respectively. The HR at 6 months was 0.97 (95% CI: 0.50–1.87; log-rank *p* = 0.915), that at 1 year was 0.99 (95% CI: 0.55–1.80; log-rank *p* = 0.975), and that at 2 years was 0.95 (95% CI: 0.55–1.64; log-rank *p* = 0.844), demonstrating non-significant, comparable risks across both groups (Figure 8 and Supplementary Figure S16) (Supplementary Table S6).

## 4. Discussion

In this large propensity-score-matched cohort of patients with venous thromboembolism, treatment with apixaban was associated with efficacy comparable to that of rivaroxaban in preventing recurrent VTE; however, a small but statistically significant increase in VTE recurrence was observed in the 6-month, 1-year, and 2-year follow-ups. Bleeding outcomes were largely comparable between groups in the overall matched cohort, with no statistically significant differences observed in major bleeding, composite major bleeding, gastrointestinal bleeding, or intracerebral hemorrhage across follow-up periods. A statistically significant reduction in non-major bleeding with apixaban was observed at the 6-month follow-up only and was not sustained at later time points. All-cause mortality was broadly comparable through 1 year; however, a statistically significant increase in mortality was observed at the 2-year follow-up among apixaban users. Subgroup analyses identified important heterogeneity in bleeding outcomes: composite major bleeding was significantly lower with apixaban among patients with obesity at the 2-year follow-up, whereas composite major bleeding and major bleeding were significantly higher with apixaban among patients with cancer at the 1-year and 2-year follow-ups. Baseline demographic, clinical, and laboratory characteristics were well balanced after PSM, supporting adequate comparability between matched cohorts. These results suggest that apixaban and rivaroxaban demonstrate broadly similar effectiveness and safety profiles in routine clinical practice, with subgroup-specific differences that warrant further prospective investigation (Figure 9).

Apixaban demonstrates dose-proportional exposure and low inter-individual variability, resulting in relatively consistent pharmacokinetic profiles across patients [18,19]. Apixaban is eliminated via multiple pathways, with approximately 27% of total clearance occurring through the renal route, whereas rivaroxaban demonstrates a higher renal contribution (~33–36%), in addition to significant fecal elimination [20,21]. Apixaban is administered twice daily, whereas rivaroxaban is administered once daily, according to their approved dosing regimens and pharmacokinetic characteristics [18]. Variability in laboratory-measured anticoagulant effects has been described among direct oral anticoagulants, while all agents demonstrate dose-dependent pharmacodynamic activity [22]. Pharmacokinetic profiles of rivaroxaban include concentration–time fluctuations associated with once-daily administration, reflecting peak and trough concentrations over the dosing interval [23]. These pharmacokinetic differences may be associated with differences in observed clinical outcomes; however, no causal relationship can be established in this observational study.

Beyond their distinct pharmacokinetic characteristics, the comparable efficacy between these agents in preventing recurrent VTE aligns with their shared mechanism as direct factor Xa inhibitors. Both medications inhibit free and clot-bound factor Xa, as well as prothrombinase activity, thereby interrupting the coagulation cascade and preventing thrombin generation [24,25]. The similar hazard ratios for VTE recurrence across multiple time points (3-month, 6-month, 1-year, and 2-year follow-ups) suggest near-equivalent efficacy overall, with small but statistically significant increases in recurrence observed at 6-month, 1-year, and 2-year follow-ups. Although the hazard ratios reached statistical significance at several time points, the absolute differences in recurrence rates between groups were small and should be interpreted cautiously in terms of clinical relevance. Subgroup analyses among patients with obesity and cancer demonstrated generally comparable recurrence rates between treatment groups, with no statistically significant differences observed. Cancer-associated thrombosis represents a high-risk clinical setting characterized by complex management decisions and variability in treatment strategies, as reflected in current guideline recommendations and clinical trial data [26,27]. These observations are consistent with large registry data from the RIETE Investigators, who have demonstrated associations between baseline clinical characteristics and both mortality and bleeding outcomes in patients with acute VTE [28,29,30]. Although no statistically significant differences were observed in the cancer subgroup, these findings should be interpreted cautiously given the complexity of cancer-associated thrombosis and potential residual confounding inherent to observational studies.

These findings are consistent with evidence from major randomized controlled trials evaluating both drugs for VTE treatment. In the CARAVAGGIO trial, Agnelli et al. demonstrated that apixaban was non-inferior to dalteparin for prevention of recurrent VTE in patients with cancer, without a statistically significant increase in major bleeding (HR 0.82, 95% CI 0.40–1.69) [31]. Similarly, the EINSTEIN-DVT and EINSTEIN-PE trials demonstrated that rivaroxaban was non-inferior to standard therapy for the treatment of venous thromboembolism, with similar or lower rates of major bleeding compared with conventional anticoagulation [32,33]. A subsequent network meta-analysis by Alexander T. Cohen et al. comparing direct oral anticoagulants found that apixaban had the most favorable bleeding profile, with significantly lower rates of major or clinically relevant non-major bleeding compared with other DOACs for VTE treatment, although our study demonstrated largely comparable bleeding outcomes between apixaban and rivaroxaban [34]. In contrast to some prior studies suggesting apixaban offers a bleeding advantage, our findings demonstrate largely comparable bleeding outcomes between apixaban and rivaroxaban in the overall matched cohort. Furthermore, cancer subgroup analyses showed comparable recurrent VTE rates between treatment groups, supporting similar effectiveness in this high-risk population. Further recent high-quality evidence further contextualizes these findings. The COBRRA trial by Castellucci et al. reported a consistent reduction in major and clinically relevant bleeding with apixaban-based strategies compared with rivaroxaban-based regimens, whereas our real-world propensity-matched analysis demonstrates no statistically significant differences in major bleeding, gastrointestinal bleeding, or intracerebral hemorrhage, suggesting attenuation of bleeding differences in routine clinical practice [13]. Similarly, the large observational study by Sun et al. (PLoS Medicine, 2025) reported similar risks of recurrent VTE and major bleeding between apixaban and rivaroxaban in cancer-associated VTE while demonstrating a lower risk of clinically relevant non-major bleeding with apixaban [35].

A large administrative database study conducted by Dawwas et al. using the US Truven Health MarketScan commercial and Medicare Supplement claims databases found that apixaban was associated with a significantly lower risk of major bleeding compared with rivaroxaban among VTE patients (HR 0.54 [95% CI 0.37–0.82]) [36]. Additionally, a meta-analysis by Fredman et al. reported significantly lower major bleeding with apixaban relative to rivaroxaban [37]. More recent real-world registry analyses have similarly demonstrated lower bleeding rates with apixaban compared with rivaroxaban across anticoagulated populations [38]. In contrast, our study found no statistically significant differences in major bleeding (composite), major bleeding, gastrointestinal bleeding, or intracerebral hemorrhage between apixaban and rivaroxaban in the overall propensity-matched cohort. A lower risk of non-major bleeding was observed with apixaban at the 6-month follow-up; however, this difference was not consistently observed at later time points. We also observed a non-significant trend toward higher intracerebral hemorrhage rates with apixaban, although confidence intervals crossed unity in all follow-up periods. All-cause mortality was generally comparable between groups through 1 year; however, a statistically significant increase in mortality was observed at the 2-year follow-up among apixaban users. Subgroup analyses revealed important heterogeneity in bleeding outcomes. Among patients with obesity, major bleeding (composite) was significantly lower with apixaban at the 2-year follow-up. Conversely, among patients with cancer, major bleeding (composite) was significantly higher with apixaban at both the 1-year and 2-year follow-ups, and major bleeding was significantly higher at 1 year. These findings should be interpreted cautiously given the observational nature of the study and the potential for residual confounding. Overall, our findings suggest that apixaban and rivaroxaban provide broadly comparable effectiveness and safety for the treatment of VTE in routine clinical practice. Although small statistically significant differences in recurrent VTE were observed at the selected time points, the absolute differences were modest. Bleeding outcomes were generally similar between groups in the overall cohort, while subgroup analyses identified potentially important differences that warrant further investigation.

### Limitations

There are several limitations to be acknowledged. First, the observational design of this study precludes any conclusions about causality, even though PSM was used to balance baseline characteristics. Second, while the TriNetX database provides comprehensive electronic health records, there remains potential for misclassification or coding errors in the documentation of VTE, comorbidities, medication use, and clinical outcomes. In particular, recurrent VTE identification relied on diagnostic coding occurring after the index event and may have included repeat encounters or persistent thrombosis despite exclusion of index events. Third, detailed clinical information, including VTE location (proximal vs. distal DVT and pulmonary embolism burden), duration of anticoagulation therapy, dose adjustments, medication adherence, switching between anticoagulants, and reasons for treatment selection, was not consistently captured, which may have affected the outcomes. Analyses were performed according to the initially prescribed anticoagulant using an intention-to-treat approach; therefore, subsequent treatment discontinuation, interruption, switching between DOACs, crossover to alternative anticoagulants, and differences in treatment persistence could not be reliably captured. Consequently, clinically relevant time-varying treatment exposures were not accounted for, and outcome events occurring during follow-up may not have reflected continued exposure to the index anticoagulant. These post-baseline treatment modifications may have introduced exposure misclassification and biased long-term comparative estimates of recurrent VTE, bleeding, and mortality, potentially attenuating or exaggerating observed differences between treatment groups. Additionally, detailed information regarding loading versus maintenance dosing, treatment discontinuation, and longitudinal treatment persistence was not reliably available within TriNetX. Consequently, analyses were performed according to the initially prescribed anticoagulant, and the impact of subsequent treatment modifications on clinical outcomes could not be assessed.

Fourth, data on provoked versus unprovoked VTE, thrombophilia testing, and long-term anticoagulation decisions were unavailable, limiting assessment of treatment appropriateness. Fifth, the higher rate of VTE recurrence in the cancer subgroup treated with apixaban may reflect surveillance bias, differences in cancer types and stages, or residual confounding related to disease severity. Accordingly, cancer subgroup analyses should be interpreted as exploratory and hypothesis-generating. Sixth, residual confounding from unmeasured variables (such as frailty, bleeding risk scores, and physician prescribing preferences) cannot be excluded. In addition, important factors including renal function severity, body weight distribution, cancer stage, VTE severity, medication adherence, dose selection, and duration of therapy may not have been fully captured within the database and could have influenced treatment allocation and clinical outcomes despite propensity score matching. Additionally, ICD-10-code-based bleeding ascertainment may underestimate clinically significant bleeding events and may not align precisely with International Society on Thrombosis and Haemostasis (ISTH) major bleeding criteria. Furthermore, ABO blood group and detailed active cancer phenotype characteristics were not uniformly available within the database and therefore could not be incorporated into the propensity-score-matching model. Inclusion of the ABO blood group in the matching algorithm resulted in a complete lack of matched patients remaining, due to substantial missingness and limited cohort overlap. Residual confounding related to these factors cannot be excluded, and proportional hazards assumptions could not be formally tested within the TriNetX platform. Finally, rare complications or adverse events may be under-reported in real-world EHR data.

## 5. Conclusions

In patients with venous thromboembolism, rates of recurrent VTE were broadly similar between apixaban- and rivaroxaban-treated patients, although a small but statistically significant increase in recurrent VTE was observed with apixaban at 6-month, 1-year, and 2-year follow-ups. These findings should be interpreted cautiously given the modest absolute differences, observational design, and potential for residual confounding. Bleeding outcomes were largely comparable between groups in the overall propensity-matched cohort, with no statistically significant differences observed in major bleeding, composite major bleeding, gastrointestinal bleeding, or intracerebral hemorrhage. A statistically significant reduction in non-major bleeding with apixaban was observed at the 6-month follow-up only and was not sustained at later time points. All-cause mortality was broadly comparable between groups through 1 year; however, a statistically significant increase in mortality was observed at the 2-year follow-up among apixaban users, a finding that warrants further investigation. Subgroup analyses revealed important heterogeneity: among patients with obesity, composite major bleeding was significantly lower with apixaban at the 2-year follow-up, whereas among patients with cancer, composite major bleeding and major bleeding were significantly higher with apixaban at the 1-year and 2-year follow-ups. These subgroup findings should be considered exploratory and hypothesis-generating given the observational nature of this study. While differences in pharmacokinetic properties, including renal clearance and dosing frequency, may contribute to the outcome differences observed, these findings do not establish causality or superiority. These findings support individualized treatment selection based on bleeding risk, renal function, cancer status, and patient-specific factors and underscore the need for prospective randomized controlled trials with longer follow-ups to further evaluate the comparative safety and effectiveness of apixaban and rivaroxaban in a diverse VTE population.

## Figures and Tables

**Table 1:** Baseline characteristics.

Characteristic	Apixaban and VTE Group (N = 26,796)	Rivaroxaban and VTE Group (N = 8793)	Std Diff.	Apixaban and VTE Group (N = 8247)	Rivaroxaban and VTE Group (N = 8247)	Std Diff.
	Before PSM			After PSM		
Demographics						
Age at Index (yrs)	62.7 ± 16.9	56.8 ± 18.1	0.337	58.3 ± 18.0	57.3 ± 18.1	0.054
0–40 years	3056 (11.40%)	1669 (19.00%)	0.212	1509 (18.30%)	1495 (18.10%)	0.004
40–65 years	9989 (37.30%)	3806 (43.30%)	0.123	3442 (41.70%)	3547 (43.00%)	0.026
65–75 years	6484 (24.20%)	1713 (19.50%)	0.114	1671 (20.30%)	1637 (19.80%)	0.01
75–0 years	7267 (27.10%)	1605 (18.30%)	0.213	1625 (19.70%)	1568 (19.00%)	0.017
Male	14,495 (54.10%)	4795 (54.50%)	0.009	4471 (54.20%)	4531 (54.90%)	0.015
Female	12,293 (45.90%)	3996 (45.40%)	0.009	3773 (45.70%)	3714 (45.00%)	0.014
White	19,101 (71.30%)	5775 (65.70%)	0.121	5485 (66.50%)	5585 (67.70%)	0.026
Black or African American	3407 (12.70%)	954 (10.80%)	0.058	947 (11.50%)	943 (11.40%)	0.002
American Indian or Alaska Native	121 (0.50%)	27 (0.30%)	0.024	25 (0.30%)	27 (0.30%)	0.004
Native Hawaiian or Other Pacific Islander	66 (0.20%)	25 (0.30%)	0.007	26 (0.30%)	24 (0.30%)	0.004
Unknown Ethnicity	5498 (20.50%)	2522 (28.70%)	0.19	2040 (24.70%)	2069 (25.10%)	0.008
Not Hispanic or Latino	19,465 (72.60%)	5751 (65.40%)	0.157	5648 (68.50%)	5666 (68.70%)	0.005
Hispanic or Latino	1833 (6.80%)	520 (5.90%)	0.038	559 (6.80%)	512 (6.20%)	0.023
Asian	468 (1.70%)	195 (2.20%)	0.034	197 (2.40%)	175 (2.10%)	0.018
Comorbidities						
Hypertensive diseases	11,035 (41.20%)	2219 (25.20%)	0.344	2265 (27.50%)	2168 (26.30%)	0.027
Heart failure	3182 (11.90%)	531 (6.00%)	0.205	538 (6.50%)	516 (6.30%)	0.011
Chronic ischemic heart disease	3149 (11.80%)	545 (6.20%)	0.195	588 (7.10%)	538 (6.50%)	0.024
Acute myocardial infarction	1695 (6.30%)	269 (3.10%)	0.155	294 (3.60%)	266 (3.20%)	0.019
Cerebrovascular diseases	2067 (7.70%)	341 (3.90%)	0.165	333 (4.00%)	336 (4.10%)	0.002
Atrial fibrillation and flutter	2809 (10.50%)	451 (5.10%)	0.201	452 (5.50%)	448 (5.40%)	0.002
Atherosclerosis	1018 (3.80%)	172 (2.00%)	0.11	175 (2.10%)	172 (2.10%)	0.003
Type 2 diabetes mellitus	4180 (15.60%)	841 (9.60%)	0.183	864 (10.50%)	825 (10.00%)	0.016
Type 1 diabetes mellitus	133 (0.50%)	35 (0.40%)	0.015	36 (0.40%)	35 (0.40%)	0.002
Overweight and obesity	3203 (12.00%)	674 (7.70%)	0.145	686 (8.30%)	663 (8.00%)	0.01
Cerebral infarction	1143 (4.30%)	162 (1.80%)	0.141	158 (1.90%)	159 (1.90%)	0.001
Nontraumatic intracerebral hemorrhage	221 (0.80%)	35 (0.40%)	0.055	38 (0.50%)	35 (0.40%)	0.005
Diseases of liver	1320 (4.90%)	245 (2.80%)	0.111	247 (3.00%)	242 (2.90%)	0.004
Gastrointestinal hemorrhage, unspecified	277 (1.00%)	65 (0.70%)	0.031	70 (0.80%)	65 (0.80%)	0.007
Hemorrhage, not elsewhere classified	87 (0.30%)	16 (0.20%)	0.028	21 (0.30%)	16 (0.20%)	0.013
Malaise and fatigue	2058 (7.70%)	418 (4.80%)	0.121	412 (5.00%)	410 (5.00%)	0.001
Coagulation defects, purpura and other hemorrhagic conditions	2144 (8.00%)	484 (5.50%)	0.1	483 (5.90%)	481 (5.80%)	0.001
Chronic kidney disease (CKD)	2584 (9.60%)	257 (2.90%)	0.28	330 (4.00%)	256 (3.10%)	0.048
Other anemias	2672 (10.00%)	475 (5.40%)	0.172	495 (6.00%)	468 (5.70%)	0.014
Personal history of nicotine dependence	3319 (12.40%)	616 (7.00%)	0.183	624 (7.60%)	613 (7.40%)	0.005
Pulmonary embolism	12,923 (48.20%)	3918 (44.60%)	0.074	3584 (43.50%)	3492 (42.30%)	0.023
Acute embolism and thrombosis of deep veins of lower extremity	8695 (32.40%)	2287(26.00%)	0.142	2310 (28.00%)	2209 (26.80%)	0.027
Acute embolism and thrombosis of veins of upper extremity	1275 (4.80%)	316 (3.60%)	0.058	308 (3.70%)	312 (3.80%)	0.003
Medications						
Platelet Aggregation Inhibitors	5003 (18.70%)	1183 (13.50%)	0.142	1144 (13.90%)	1142 (13.80%)	0.001
Non-Steroidal Anti-Inflammatory Analgesics	1330 (5.00%)	459 (5.20%)	0.012	453 (5.50%)	450 (5.50%)	0.002
Non-Opioid Analgesics	14,035 (52.40%)	3755 (42.70%)	0.195	3631 (44.00%)	3575 (43.30%)	0.014
Adrenal Corticosteroids	6317 (23.60%)	1626 (18.50%)	0.125	1540 (18.70%)	1545 (18.70%)	0.002
Beta Blockers/Related	6017 (22.50%)	1318 (15.00%)	0.192	1319 (16.00%)	1263 (15.30%)	0.019
Calcium Channel Blockers	3545 (13.20%)	739 (8.40%)	0.156	709 (8.60%)	696 (8.40%)	0.006
Angiotensin II Inhibitor	2170 (8.10%)	553 (6.30%)	0.07	501 (6.10%)	491 (6.00%)	0.005
Antilipemic Agents	6294 (23.50%)	1341 (15.30%)	0.21	1327 (16.10%)	1289 (15.60%)	0.013
Gastric Medications, Other	6012 (22.40%)	1748 (19.90%)	0.063	1557 (18.90%)	1550 (18.80%)	0.002
Blood Glucose Regulation Agents	6336 (23.60%)	1629 (18.50%)	0.126	1522 (18.50%)	1483 (18.00%)	0.012
Heparin	10,931 (40.80%)	2479 (28.20%)	0.267	2450 (29.70%)	2395 (29.00%)	0.015
Enoxaparin	6842 (25.50%)	2669 (30.40%)	0.108	2323 (28.20%)	2259 (27.40%)	0.017
Laboratory Values						
Creatinine, mg/dL	1.1 ± 1.9	1.0 ± 2.7	0.035	1.1 ± 3.5	1.0 ± 2.5	0.032
Creatinine, 0–0	18,218 (68.00%)	5035 (57.30%)	0.223	4810 (58.30%)	4603 (55.80%)	0.051
Hemoglobin, g/dL	12.1 ± 2.4	12.5 ± 2.3	0.205	12.3 ± 2.4	12.5 ± 2.3	0.109
Hemoglobin, 0–0 g/dL	17,451 (65.10%)	4983 (56.70%)	0.174	4688 (56.80%)	4532 (55.00%)	0.038
Platelets, 10^3^/µL	234.9 ± 98.5	233.4 ± 94.3	0.015	238.7 ± 97.7	233.1 ± 95.3	0.058
Platelets, 0–0	19,045 (71.10%)	5287 (60.10%)	0.232	5023 (60.90%)	4839 (58.70%)	0.046
Alanine aminotransferase, U/L	34.6 ± 68.1	33.6 ± 66.5	0.015	34.4 ± 55.0	33.7 ± 68.2	0.01
Alanine aminotransferase, 0–0	14,774 (55.10%)	3659 (41.60%)	0.273	3542 (42.90%)	3388 (41.10%)	0.038
Aspartate aminotransferase, U/L	35.6 ± 62.7	33.8 ± 69.9	0.027	35.1 ± 52.7	34.2 ± 71.9	0.014
Aspartate aminotransferase, 0–0	14,495 (54.10%)	3484 (39.60%)	0.293	3395 (41.20%)	3268 (39.60%)	0.031
Bilirubin.total, mg/dL	0.7 ± 0.7	0.8 ± 1.7	0.037	0.7 ± 0.6	0.8 ± 1.6	0.052
Bilirubin.total, 0–0	13,704 (51.10%)	3157 (35.90%)	0.311	3121 (37.80%)	3013 (36.50%)	0.027
Albumin, g/dL	3.5 ± 0.7	3.6 ± 0.7	0.178	3.5 ± 0.7	3.6 ± 0.7	0.096
Albumin, 0–0	14,652 (54.70%)	3194 (36.30%)	0.375	3214 (39.00%)	3161 (38.30%)	0.013
Activated partial thromboplastin time (aPTT), s	42.6 ± 26.3	38.5 ± 23.6	0.163	40.4 ± 25.1	39.7 ± 23.7	0.029
Activated partial thromboplastin time (aPTT), 0–0	11,343 (42.30%)	3363 (38.20%)	0.083	3160 (38.30%)	3035 (36.80%)	0.031
INR	1.2 ± 0.3	1.2 ± 0.3	0.006	1.2 ± 0.2	1.2 ± 0.3	0.072
INR, 0–0	12,381 (46.20%)	3660 (41.60%)	0.092	3472 (42.10%)	3318 (40.20%)	0.038
Prothrombin time (PT), s	13.5 ± 3.4	12.7 ± 4.4	0.191	13.3 ± 3.2	13.1 ± 4.1	0.031
Prothrombin time (PT) 0–0, s	9276 (34.60%)	2624 (29.80%)	0.102	2551 (30.90%)	2357 (28.60%)	0.051
Cholesterol, mg/dL	166.5 ± 47.9	174.6 ± 48.4	0.17	172.3 ± 44.7	173.5 ± 48.4	0.026
Cholesterol, 0–0	5053 (18.90%)	1117 (12.70%)	0.169	1074 (13.00%)	1047 (12.70%)	0.01
Cholesterol in LDL, mg/dL	95.7 ± 38.9	101.6 ± 38.3	0.153	100.9 ± 36.6	101.0 ± 38.4	0.005
Cholesterol in LDL, 0–0	4988 (18.60%)	1068 (12.10%)	0.18	1038 (12.60%)	1014 (12.30%)	0.009
Cholesterol in HDL, mg/dL	47.1 ± 16.9	48.6 ± 16.7	0.089	48.3 ± 16.8	48.3 ± 16.6	0.001
Cholesterol in HDL, 0–0	4983 (18.60%)	1073 (12.20%)	0.178	1037 (12.60%)	1017 (12.30%)	0.007
Sodium, mmol/L	138.0 ± 3.7	138.1 ± 3.7	0.022	137.9 ± 3.5	138.1 ± 3.6	0.038
Sodium, 0–0	19,253 (71.90%)	5179 (58.90%)	0.275	4998 (60.60%)	4825 (58.50%)	0.043
Potassium, mmol/L	4.0 ± 0.5	4.0 ± 0.5	0.026	4.0 ± 0.5	4.0 ± 0.5	0.039
Potassium, 0–0	18,933 (70.70%)	5200 (59.10%)	0.243	4970 (60.30%)	4801 (58.20%)	0.042
Urea nitrogen, mg/dL	18.7 ± 12.2	15.4 ± 8.1	0.315	17.0 ± 10.7	15.5 ± 8.2	0.159
Urea nitrogen, 0–0	17,954 (67.00%)	4518 (51.40%)	0.322	4544 (55.10%)	4415 (53.50%)	0.031
Phosphate, mg/dL	3.3 ± 0.9	3.3 ± 0.9	0.001	3.3 ± 0.9	3.3 ± 0.8	0.043
Phosphate, 0–0	7745 (28.90%)	1545 (17.60%)	0.271	1573 (19.10%)	1467 (17.80%)	0.033
Hemoglobin A1c/Hemoglobin.total, %	6.5 ± 1.8	6.6 ± 2.0	0.066	6.6 ± 2.0	6.7 ± 2.0	0.036
Hemoglobin A1c/Hemoglobin.total, 0–0	5231 (19.50%)	987 (11.20%)	0.232	972 (11.80%)	946 (11.50%)	0.01
BMI kg/m^2^	28.9 ± 5.9	29.0 ± 5.7	0.01	29.1 ± 6.1	29.0 ± 5.7	0.017
0–18.50 kg/m^2^	10,593 (39.50%)	2829 (32.20%)	0.05	2765 (33.50%)	2699 (32.70%)	0.005
18.50–25 kg/m^2^	263 (1.00%)	48 (0.50%)	0.095	50 (0.60%)	47 (0.60%)	0.011
25–30 kg/m^2^	2911 (10.90%)	712 (8.10%)	0.084	702 (8.50%)	678 (8.20%)	0.015
30–35 kg/m^2^	4479 (16.70%)	1204 (13.70%)	0.076	1185 (14.40%)	1141 (13.80%)	0.007
35–40 kg/m^2^	3066 (11.40%)	802 (9.10%)	0.062	791 (9.60%)	775 (9.40%)	0.001
40–0 kg/m^2^	1306 (4.90%)	318 (3.60%)	0.029	309 (3.70%)	310 (3.80%)	0.011
Body weight lb	183.5 ± 34.9	184.8 ± 34.9	0.037	184.2 ± 34.6	185.1 ± 34.8	0.027
0–0	12,873 (48.00%)	3273 (37.20%)	0.22	3178 (38.50%)	3127 (37.90%)	0.013

**Table 2:** Clinical outcomes at multiple follow-up intervals in the overall propensity-score-matched acute venous thromboembolism (VTE) population.

Outcome	Apixaban and VTE Group (n/N)	Rivaroxaban and VTE Group (n/N)	HR (95% CI)	Log-Rank *p* Value	ARD (95% CI)
Primary Outcome					
Recurrent VTE					
At 3 Months *	1826/8247	1836/8247	1.02 (0.95–1.09)	0.600	−0.001 (−0.014 to 0.011)
At 6 Months *	2457/8247	2381/8247	1.07 (1.01–1.13)	0.016	0.009 (−0.005 to 0.023)
At 1 Year *	2715/8247	2679/8247	1.07 (1.01–1.12)	0.019	0.004 (−0.010 to 0.019)
At 2 Years * ^[1]^	4303/12,035	4312/12,035	1.08 (1.04–1.13)	0.000	−0.001 (−0.013 to 0.011)
Secondary Outcome					
All-Cause Mortality					
At 3 Months	243/8227	222/8224	1.11 (0.93–1.34)	0.247	0.003 (−0.003 to 0.008)
At 6 Months	325/8294	299/8296	1.12 (0.95–1.31)	0.171	0.003 (−0.003 to 0.009)
At 1 Year	412/8227	381/8224	1.14 (0.99–1.31)	0.064	0.004 (−0.003 to 0.010)
At 2 Years	492/8072	449/8074	1.20 (1.06–1.37)	0.005	0.005 (−0.002 to 0.013)
Major Bleeding (Composite)					
At 3 Months	264/7322	255/7535	1.09 (0.91–1.29)	0.354	0.002 (−0.004 to 0.008)
At 6 Months	316/7400	332/7604	1.00 (0.86–1.17)	0.990	−0.001 (−0.007 to 0.006)
At 1 Year	399/7322	417/7535	1.00 (0.86–1.17)	0.620	−0.001 (−0.008–0.006)
At 2 Years	479/7207	521/7410	1.03 (0.91–1.17)	0.641	−0.004 (−0.012 to 0.004)
Non-Major Bleeding					
At 3 Months	125/7884	136/7966	0.94 (0.74–1.20)	0.635	−0.001 (−0.005 to 0.003)
At 6 Months	139/7963	182/8036	0.79 (0.63–0.98)	0.033	−0.005 (−0.010 to −0.001)
At 1 Year	203/7884	226/7966	0.96 (0.79–1.15)	0.634	−0.003 (−0.008 to 0.002)
At 2 Years	249/7731	283/7820	0.98 (0.82–1.16)	0.780	−0.004 (−0.010 to 0.002)
Major Bleeding					
At 3 Months	135/7658	121/7809	1.16 (0.91–1.48)	0.240	0.002 (−0.002 to 0.006)
At 6 Months	174/7743	153/7880	1.19 (0.96–1.48)	0.120	0.003 (−0.001 to 0.008)
At 1 Year	211/7658	193/7809	1.17 (0.97–1.43)	0.109	0.003 (−0.002 to 0.008)
At 2 Years	258/7559	249/7680	1.15 (0.97–1.37)	0.113	0.002 (−0.004 to 0.007)
ICH					
At 3 Months	28/8136	18/8160	1.59 (0.88–2.88)	0.119	0.001 (0.000–0.003)
At 6 Months	38/8203	28/8232	1.41 (0.86–2.29)	0.168	0.001 (−0.001–0.003)
At 1 Year	43/8136	37/8160	1.24 (0.80–1.92)	0.345	0.001 (−0.001–0.003)
At 2 Years	50/7994	48/8016	1.15 (0.77–1.71)	0.487	0.000 (−0.002–0.003)
GI Bleeding					
At 3 Months	52/8140	53/8148	1.00 (0.68–1.46)	0.988	0.000 (−0.003–0.002)
At 6 Months	55/8211	66/8219	0.85 (0.60–1.22)	0.385	−0.001 (−0.004–0.001)
At 1 Year	77/8140	84/8148	0.96 (0.71–1.31)	0.812	−0.001 (−0.004–0.002)
At 2 Years	100/7997	107/8001	1.03 (0.78–1.35)	0.834	−0.001 (−0.004–0.003)

ARD = absolute risk difference; CI = confidence interval; HR = hazard ratio; n = population with outcome; N = total population of cohort. Denominators vary across outcomes due to outcome-specific cohort definitions, eligibility criteria, and variable data availability within the TriNetX database. * Outcome with a lag window of 30 days (from day 1 to day 30). ^[1]^ Outcome was assessed at a different date 18 June 2026.

## Data Availability

The data that support the findings of this study are available from the TriNetX Research Network. Due to licensing restrictions and data use agreements, the raw data are not publicly available. Access to the TriNetX platform can be gained through institutional subscription. The aggregate data supporting the findings of this study may be available from the corresponding author upon reasonable request and with permission from TriNetX.

## Supplementary Materials

The following supporting information can be downloaded at https://www.mdpi.com/article/10.3390/jcm15145410/s1. Figure S1: Propensity Score Matching; Figure S2: Recurrent VTE, Subgrouped by Weight > 120 Kg (Obese) at 1-Year; Figure S3: Recurrent VTE, Subgrouped by Cancer; Figure S4: All-Cause Mortality; Figure S5: All-Cause Mortality, Subgrouped by Weight > 120 Kg (Obese); Figure S6: All-Cause Mortality, Subgrouped by Cancer; Figure S7: Major Bleeding (Composite); Figure S8: Major Bleeding (Composite), Subgrouped by Weight > 120 Kg (Obese); Figure S9: Major Bleeding (Composite), Subgrouped by Cancer; Figure S10: Non-Major Bleed; Figure S11: Non-Major Bleed, Subgrouped by Cancer; Figure S12: Major Bleeding; Figure S13: Major Bleeding, Subgrouped by Cancer; Figure S14: ICH; Figure S15: GI Bleed; Figure S16: GI Bleed, Subgrouped by Cancer; Table S1: Criteria for Cohort Apixaban and VTE; Table S2: Criteria for Cohort Rivaroxaban and VTE; Table S3: Outcome Definitions; Table S4: ICD Codes; Table S5: Outcomes, Subgrouped by Weight > 120 Kg (Obese); Table S6: Outcomes, Subgrouped by Cancer; Table S7: Sensitivity Analyses for Recurrent VTE.
