# Supplementary material for: Comparison of the Safety and Effectiveness of Apixaban Versus Rivaroxaban in Acute Venous Thromboembolism: A Propensity-Matched Real-World TriNetX Study with Obesity and Cancer Subgroup Analyses
Source: J Clin Med. 2026 Jul 10;15(14):5410. doi: 10.3390/jcm15145410 (PMC13412796; doi:10.3390/jcm15145410)
Supplement: Supplementary file 1 [file jcm-15-05410-s001.zip › jcm-4344657-supplementary.pdf]

Supplementary Table S1. Criteria for Cohort Apixaban and VTE

**Ungrouped terms**				
must have		demographics	Age	Age (at least 18 years (most recent occurrence))
**Group 1**				
**Group 1A Xa Inhibitor**				
must have		medication	NLM:RXNORM:1364430	apixaban
cannot have		diagnosis	UMLS:ICD10CM:Z95.2	Presence of prosthetic heart valve
	or	diagnosis	UMLS:ICD10CM:O00-O9A	Pregnancy, childbirth and the puerperium
	or	laboratory	TNX:9081	Body weight (at most 110.00 [lb_av])
	or	laboratory	TNX:9081	Body weight (at least 265.00 [lb_av])
	or	diagnosis	UMLS:ICD10CM:K72	Hepatic failure, not elsewhere classified
	or	diagnosis	UMLS:ICD10CM:K70.4	Alcoholic hepatic failure
	or	medication	NLM:RXNORM:1114195	rivaroxaban
	or	medication	NLM:RXNORM:1037042	dabigatran etexilate
	or	medication	NLM:RXNORM:1546356	dabigatran
	or	medication	NLM:RXNORM:1599538	edoxaban
	or	laboratory	UMLS:LNC:35591-7	Creatinine renal clearance predicted by Cockcroft-Gault formula (at most 30.00 mL/min)

date constraint		This group occurred before 3 months ago		
event relationship		The first instance of Acute VTE occurred within 7 days on or before the first instance of Xa Inhibitor		
**Group 1B Acute VTE**				
must have	any of	diagnosis	UMLS:ICD10CM:I26	Pulmonary embolism
		diagnosis	UMLS:ICD10CM:I82.6	Acute embolism and thrombosis of veins of upper extremity
		diagnosis	UMLS:ICD10CM:I82.4	Acute embolism and thrombosis of deep veins of lower extremity
	and any of	visit	UMLS:HL7V3.0:VisitType:EMER	Visit: Emergency
		visit	UMLS:HL7V3.0:VisitType:ACUTE	Visit: Inpatient Acute
		visit	UMLS:HL7V3.0:VisitType:IMP	Visit: Inpatient Encounter
		visit	UMLS:HL7V3.0:VisitType:NONAC	Visit: Inpatient Non-acute
**Group 2**				
**Group 2A Xa Inhibitor**				
must have		medication	NLM:RXNORM:1364430	apixaban
cannot have		diagnosis	UMLS:ICD10CM:Z95.2	Presence of prosthetic heart valve
	or	diagnosis	UMLS:ICD10CM:O00-O9A	Pregnancy, childbirth and the puerperium

	or	laboratory	TNX:9081	Body weight (at most 110.00 [lb_av])
	or	laboratory	TNX:9081	Body weight (at least 265.00 [lb_av])
	or	diagnosis	UMLS:ICD10CM:K72	Hepatic failure, not elsewhere classified
	or	diagnosis	UMLS:ICD10CM:K70.4	Alcoholic hepatic failure
	or	medication	NLM:RXNORM:1037042	dabigatran etexilate
	or	medication	NLM:RXNORM:1114195	rivaroxaban
	or	medication	NLM:RXNORM:1546356	dabigatran
	or	medication	NLM:RXNORM:1599538	edoxaban
	or	laboratory	UMLS:LNC:35591-7	Creatinine renal clearance predicted by Cockcroft-Gault formula (at most 30.00 mL/min)
	date constraint	This group occurred before 3 months ago		
	event relationship	Any instance of Washout of DOAC occurred within 1 year and 1 day before the first instance of Xa Inhibitor		
**Group 2B Washout of DOAC**				
cannot have		medication	NLM:RXNORM:1364430	apixaban
	or	medication	NLM:RXNORM:1114195	rivaroxaban
	or	medication	NLM:RXNORM:1546356	dabigatran
	or	medication	NLM:RXNORM:1599538	edoxaban
	or	medication	NLM:RXNORM:1037042	dabigatran etexilate
**Group 3 or Group 4 must be present**				

Group 3				
Group 3A Xa Inhibitor				
must have		medication	NLM:RXNORM:1364430	apixaban
cannot have		diagnosis	UMLS:ICD10CM:Z95.2	Presence of prosthetic heart valve
	or	diagnosis	UMLS:ICD10CM:O00-O9A	Pregnancy, childbirth and the puerperium
	or	laboratory	TNX:9081	Body weight (at most 110.00 [lb_av])
	or	laboratory	TNX:9081	Body weight (at least 265.00 [lb_av])
	or	diagnosis	UMLS:ICD10CM:K72	Hepatic failure, not elsewhere classified
	or	diagnosis	UMLS:ICD10CM:K70.4	Alcoholic hepatic failure
	or	medication	NLM:RXNORM:1037042	dabigatran etexilate
	or	medication	NLM:RXNORM:1114195	rivaroxaban
	or	medication	NLM:RXNORM:1546356	dabigatran
	or	medication	NLM:RXNORM:1599538	edoxaban
	or	laboratory	UMLS:LNC:35591-7	Creatinine renal clearance predicted by Cockcroft-Gault formula (at most 30.00 mL/min)
date constraint		This group occurred before 3 months ago		
event relationship		The first instance of Cancer Exclusion occurred within 1 year on or before the first instance of Xa Inhibitor		
Group 3B Cancer Exclusion				

cannot have		diagnosis	UMLS:ICD10CM:C7A-C7A	Malignant neuroendocrine tumors (C7A)
	or	diagnosis	UMLS:ICD10CM:C81-C96	Malignant neoplasms of lymphoid, hematopoietic and related tissue
	or	diagnosis	UMLS:ICD10CM:C73-C75	Malignant neoplasms of thyroid and other endocrine glands
	or	diagnosis	UMLS:ICD10CM:C69-C72	Malignant neoplasms of eye, brain and other parts of central nervous system
	or	diagnosis	UMLS:ICD10CM:C64-C68	Malignant neoplasms of urinary tract
	or	diagnosis	UMLS:ICD10CM:C60-C63	Malignant neoplasms of male genital organs
	or	diagnosis	UMLS:ICD10CM:C50-C50	Malignant neoplasms of breast (C50)
	or	diagnosis	UMLS:ICD10CM:C51-C58	Malignant neoplasms of female genital organs
	or	diagnosis	UMLS:ICD10CM:C43-C44	Melanoma and other malignant neoplasms of skin
	or	diagnosis	UMLS:ICD10CM:C45-C49	Malignant neoplasms of mesothelial and soft tissue
	or	diagnosis	UMLS:ICD10CM:C40-C41	Malignant neoplasms of bone and articular cartilage
	or	diagnosis	UMLS:ICD10CM:C30-C39	Malignant neoplasms of respiratory and intrathoracic organs

	or	diagnosis	UMLS:ICD10CM:C15-C26	Malignant neoplasms of digestive organs
	or	diagnosis	UMLS:ICD10CM:C00-C14	Malignant neoplasms of lip, oral cavity and pharynx
**Group 4**				
**Group 4A Xa Inhibitor**				
must have		medication	NLM:RXNORM:1364430	apixaban
cannot have		diagnosis	UMLS:ICD10CM:Z95.2	Presence of prosthetic heart valve
	or	diagnosis	UMLS:ICD10CM:O00-O9A	Pregnancy, childbirth and the puerperium
	or	laboratory	TNX:9081	Body weight (at most 110.00 [lb_av])
	or	medication	NLM:RXNORM:1114195	rivaroxaban
	or	laboratory	TNX:9081	Body weight (at least 265.00 [lb_av])
	or	diagnosis	UMLS:ICD10CM:K72	Hepatic failure, not elsewhere classified
	or	diagnosis	UMLS:ICD10CM:K70.4	Alcoholic hepatic failure
	or	medication	NLM:RXNORM:1037042	dabigatran etexilate
	or	medication	NLM:RXNORM:1546356	dabigatran
	or	medication	NLM:RXNORM:1599538	edoxaban
	or	laboratory	UMLS:LNC:35591-7	Creatinine renal clearance predicted by Cockcroft-Gault formula (at most 30.00 mL/min)

date constraint		This group occurred before 3 months ago		
event relationship		Any instance of Metastatic Cancer occurred on or before the first instance of Xa Inhibitor		
**Group 4B Metastatic Cancer**				
cannot have		diagnosis	UMLS:ICD10CM:C7B-C7B	Secondary neuroendocrine tumors (C7B)
	or	diagnosis	UMLS:ICD10CM:C76-C80	Malignant neoplasms of ill-defined, other secondary and unspecified sites

Supplementary Table S2. Criteria for Cohort Rivaroxaban and VTE

**Ungrouped terms**				
must have		demographics	Age	Age (at least 18 years (most recent occurrence))
**Group 1**				
**Group 1A Xa Inhibitor**				
must have		medication	NLM:RXNORM:1114195	rivaroxaban
cannot have		diagnosis	UMLS:ICD10CM:Z95.2	Presence of prosthetic heart valve
	or	diagnosis	UMLS:ICD10CM:O00-O9A	Pregnancy, childbirth and the puerperium
	or	laboratory	TNX:9081	Body weight (at most 110.00 [lb_av])
	or	laboratory	TNX:9081	Body weight (at least 265.00 [lb_av])
	or	diagnosis	UMLS:ICD10CM:K72	Hepatic failure, not elsewhere classified
	or	diagnosis	UMLS:ICD10CM:K70.4	Alcoholic hepatic failure
	or	medication	NLM:RXNORM:1037042	dabigatran etexilate
	or	medication	NLM:RXNORM:1364430	apixaban
	or	medication	NLM:RXNORM:1546356	dabigatran
	or	medication	NLM:RXNORM:1599538	edoxaban
	or	laboratory	UMLS:LNC:35591-7	Creatinine renal clearance predicted by Cockcroft-Gault formula (at most 30.00 mL/min)

date constraint		This group occurred before 3 months ago		
event relationship		The first instance of Acute VTE occurred within 7 days on or before the first instance of Xa Inhibitor		
**Group 1B Acute VTE**				
must have	any of	diagnosis	UMLS:ICD10CM:I26	Pulmonary embolism
		diagnosis	UMLS:ICD10CM:I82.6	Acute embolism and thrombosis of veins of upper extremity
		diagnosis	UMLS:ICD10CM:I82.4	Acute embolism and thrombosis of deep veins of lower extremity
	and any of	visit	UMLS:HL7V3.0:VisitType:EMER	Visit: Emergency
		visit	UMLS:HL7V3.0:VisitType:ACUTE	Visit: Inpatient Acute
		visit	UMLS:HL7V3.0:VisitType:IMP	Visit: Inpatient Encounter
		visit	UMLS:HL7V3.0:VisitType:NONAC	Visit: Inpatient Non-acute
**Group 2**				
**Group 2A Xa Inhibitor**				
must have		medication	NLM:RXNORM:1114195	rivaroxaban
cannot have		diagnosis	UMLS:ICD10CM:Z95.2	Presence of prosthetic heart valve
	or	diagnosis	UMLS:ICD10CM:O00-O9A	Pregnancy, childbirth and the puerperium
	or	laboratory	TNX:9081	Body weight (at most 110.00 [lb_av])

	or	laboratory	TNX:9081	Body weight (at least 265.00 [lb_av])
	or	diagnosis	UMLS:ICD10CM:K72	Hepatic failure, not elsewhere classified
	or	diagnosis	UMLS:ICD10CM:K70.4	Alcoholic hepatic failure
	or	medication	NLM:RXNORM:1037042	dabigatran etexilate
	or	medication	NLM:RXNORM:1364430	apixaban
	or	medication	NLM:RXNORM:1546356	dabigatran
	or	medication	NLM:RXNORM:1599538	edoxaban
	or	laboratory	UMLS:LNC:35591-7	Creatinine renal clearance predicted by Cockcroft-Gault formula (at most 30.00 mL/min)
date constraint		This group occurred before 3 months ago		
event relationship		Any instance of Washout of DOAC occurred within 1 year and 1 day before the first instance of Xa Inhibitor		
**Group 2B Washout of DOAC**				
cannot have		medication	NLM:RXNORM:1364430	apixaban
	or	medication	NLM:RXNORM:1114195	rivaroxaban
	or	medication	NLM:RXNORM:1546356	dabigatran
	or	medication	NLM:RXNORM:1599538	edoxaban
	or	medication	NLM:RXNORM:1037042	dabigatran etexilate
**Group 3 or Group 4 must be present**				
**Group 3**				

**Group 3A Xa Inhibitor**				
must have		medication	NLM:RXNORM:1114195	rivaroxaban
cannot have		diagnosis	UMLS:ICD10CM:Z95.2	Presence of prosthetic heart valve
	or	diagnosis	UMLS:ICD10CM:O00-O9A	Pregnancy, childbirth and the puerperium
	or	laboratory	TNX:9081	Body weight (at most 110.00 [lb_av])
	or	laboratory	TNX:9081	Body weight (at least 265.00 [lb_av])
	or	diagnosis	UMLS:ICD10CM:K72	Hepatic failure, not elsewhere classified
	or	diagnosis	UMLS:ICD10CM:K70.4	Alcoholic hepatic failure
	or	medication	NLM:RXNORM:1037042	dabigatran etexilate
	or	medication	NLM:RXNORM:1364430	apixaban
	or	medication	NLM:RXNORM:1546356	dabigatran
	or	medication	NLM:RXNORM:1599538	edoxaban
	or	laboratory	UMLS:LNC:35591-7	Creatinine renal clearance predicted by Cockcroft-Gault formula (at most 30.00 mL/min)
date constraint		This group occurred before 3 months ago		
event relationship		The first instance of Cancer Exclusion occurred within 1 year on or before the first instance of Xa Inhibitor		
**Group 3B Cancer Exclusion**				
cannot have		diagnosis	UMLS:ICD10CM:C7A-C7A	Malignant neuroendocrine tumors (C7A)

	or	diagnosis	UMLS:ICD10CM:C81-C96	Malignant neoplasms of lymphoid, hematopoietic and related tissue
	or	diagnosis	UMLS:ICD10CM:C73-C75	Malignant neoplasms of thyroid and other endocrine glands
	or	diagnosis	UMLS:ICD10CM:C69-C72	Malignant neoplasms of eye, brain and other parts of central nervous system
	or	diagnosis	UMLS:ICD10CM:C64-C68	Malignant neoplasms of urinary tract
	or	diagnosis	UMLS:ICD10CM:C60-C63	Malignant neoplasms of male genital organs
	or	diagnosis	UMLS:ICD10CM:C50-C50	Malignant neoplasms of breast (C50)
	or	diagnosis	UMLS:ICD10CM:C51-C58	Malignant neoplasms of female genital organs
	or	diagnosis	UMLS:ICD10CM:C43-C44	Melanoma and other malignant neoplasms of skin
	or	diagnosis	UMLS:ICD10CM:C45-C49	Malignant neoplasms of mesothelial and soft tissue
	or	diagnosis	UMLS:ICD10CM:C40-C41	Malignant neoplasms of bone and articular cartilage
	or	diagnosis	UMLS:ICD10CM:C30-C39	Malignant neoplasms of respiratory and intrathoracic organs
	or	diagnosis	UMLS:ICD10CM:C15-C26	Malignant neoplasms of digestive organs

	or	diagnosis	UMLS:ICD10CM:C00-C14	Malignant neoplasms of lip, oral cavity and pharynx
**Group 4**				
**Group 4A Xa Inhibitor**				
must have		medication	NLM:RXNORM:1114195	rivaroxaban
cannot have		diagnosis	UMLS:ICD10CM:Z95.2	Presence of prosthetic heart valve
	or	diagnosis	UMLS:ICD10CM:O00-O9A	Pregnancy, childbirth and the puerperium
	or	laboratory	TNX:9081	Body weight (at most 110.00 [lb_av])
	or	laboratory	TNX:9081	Body weight (at least 265.00 [lb_av])
	or	diagnosis	UMLS:ICD10CM:K72	Hepatic failure, not elsewhere classified
	or	diagnosis	UMLS:ICD10CM:K70.4	Alcoholic hepatic failure
	or	medication	NLM:RXNORM:1037042	dabigatran etexilate
	or	medication	NLM:RXNORM:1364430	apixaban
	or	medication	NLM:RXNORM:1546356	dabigatran
	or	medication	NLM:RXNORM:1599538	edoxaban
	or	laboratory	UMLS:LNC:35591-7	Creatinine renal clearance predicted by Cockcroft-Gault formula (at most 30.00 mL/min)
date constraint	This group occurred before 3 months ago			
event relationship	Any instance of Metastatic Cancer occurred on or before the first instance of Xa Inhibitor			

**Group 4B Metastatic Cancer**				
cannot have		diagnosis	UMLS:ICD10CM:C7B-C7B	Secondary neuroendocrine tumors (C7B)
	or	diagnosis	UMLS:ICD10CM:C76-C80	Malignant neoplasms of ill-defined, other secondary and unspecified sites

Supplementary Table S3. Outcome Definitions

**All-Cause Mortaity**			
**Outcome definition**			
	Demographics	Deceased	Deceased
**Settings for the performed analyses**			
	Risk analysis		excluding patients with outcome prior to the time window
	Kaplan - Meier survival analysis		excluding patients with outcome prior to the time window
**Major Bleeding (Composite)**			
**Outcome definition**			
	Diagnosis	UMLS:ICD10CM:I60	Nontraumatic subarachnoid hemorrhage
	Diagnosis	UMLS:ICD10CM:I61	Nontraumatic intracerebral hemorrhage
	Diagnosis	UMLS:ICD10CM:I62	Other and unspecified nontraumatic intracranial hemorrhage
	Diagnosis	UMLS:ICD10CM:K25.0	Acute gastric ulcer with hemorrhage
	Diagnosis	UMLS:ICD10CM:K25.4	Chronic or unspecified gastric ulcer with hemorrhage
	Diagnosis	UMLS:ICD10CM:K26.4	Chronic or unspecified duodenal ulcer with hemorrhage
	Diagnosis	UMLS:ICD10CM:K26.0	Acute duodenal ulcer with hemorrhage
	Diagnosis	UMLS:ICD10CM:M25.0	Hemarthrosis
	Diagnosis	UMLS:ICD10CM:H35.6	Retinal hemorrhage
	Diagnosis	UMLS:ICD10CM:K92.2	Gastrointestinal hemorrhage, unspecified
	Diagnosis	UMLS:ICD10CM:D62	Acute posthemorrhagic anemia
	Procedure	UMLS:CPT:36430	Transfusion, blood or blood components
	Diagnosis	UMLS:ICD10CM:R58	Hemorrhage, not elsewhere classified
	Diagnosis	UMLS:ICD10CM:R31	Hematuria
	Diagnosis	UMLS:ICD10CM:N93.9	Abnormal uterine and vaginal bleeding, unspecified
**Settings for the performed analyses**			
	Kaplan - Meier survival analysis		excluding patients with outcome prior to the time window
	Risk analysis		excluding patients with outcome prior to the time window
	Number of instances analysis		excluding patients with outcome prior to the time window<br>excluding patients with zero outcomes<br>counts are grouped by date
**Recurrent VTE**			
**Outcome definition**			
	Diagnosis	UMLS:ICD10CM:I26	Pulmonary embolism
	Diagnosis	UMLS:ICD10CM:I82.4	Acute embolism and thrombosis of deep veins of lower extremity
	Diagnosis	UMLS:ICD10CM:I82.6	Acute embolism and thrombosis of veins of upper extremity
**Settings for the performed analyses**			
	Risk analysis		including patients with outcome prior to the time window
	Kaplan - Meier survival analysis		including patients with outcome prior to the time window
	Number of instances analysis		including patients with outcome prior to the time window<br>excluding patients with zero outcomes<br>counts are grouped by date
**Non-Major Bleed**			
**Outcome definition**			
	Diagnosis	UMLS:ICD10CM:R58	Hemorrhage, not elsewhere classified

	Diagnosis	UMLS:ICD10CM:N93.9	Abnormal uterine and vaginal bleeding, unspecified
	Diagnosis	UMLS:ICD10CM:R31	Hematuria
**Settings for the performed analyses**			
	Kaplan - Meier survival analysis		excluding patients with outcome prior to the time window
	Number of instances analysis		excluding patients with outcome prior to the time window<br>excluding patients with zero outcomes<br>counts are grouped by date
	Risk analysis		excluding patients with outcome prior to the time window
**Major Bleeding**			
**Outcome definition**			
	Diagnosis	UMLS:ICD10CM:I61	Nontraumatic intracerebral hemorrhage
	Diagnosis	UMLS:ICD10CM:I62	Other and unspecified nontraumatic intracranial hemorrhage
	Diagnosis	UMLS:ICD10CM:I60	Nontraumatic subarachnoid hemorrhage
	Diagnosis	UMLS:ICD10CM:K25.0	Acute gastric ulcer with hemorrhage
	Diagnosis	UMLS:ICD10CM:K25.4	Chronic or unspecified gastric ulcer with hemorrhage
	Diagnosis	UMLS:ICD10CM:K26.4	Chronic or unspecified duodenal ulcer with hemorrhage
	Diagnosis	UMLS:ICD10CM:K26.0	Acute duodenal ulcer with hemorrhage
	Diagnosis	UMLS:ICD10CM:M25.0	Hemarthrosis
	Diagnosis	UMLS:ICD10CM:H35.6	Retinal hemorrhage
	Diagnosis	UMLS:ICD10CM:D62	Acute posthemorrhagic anemia
	Diagnosis	UMLS:ICD10CM:K92.2	Gastrointestinal hemorrhage, unspecified
**Settings for the performed analyses**			
	Kaplan - Meier survival analysis		excluding patients with outcome prior to the time window
	Risk analysis		excluding patients with outcome prior to the time window
	Number of instances analysis		excluding patients with outcome prior to the time window<br>excluding patients with zero outcomes<br>counts are grouped by date
**ICH**			
**Outcome definition**			
	Diagnosis	UMLS:ICD10CM:I61	Nontraumatic intracerebral hemorrhage
	Diagnosis	UMLS:ICD10CM:I62	Other and unspecified nontraumatic intracranial hemorrhage
	Diagnosis	UMLS:ICD10CM:I60	Nontraumatic subarachnoid hemorrhage
**Settings for the performed analyses**			
	Kaplan - Meier survival analysis		excluding patients with outcome prior to the time window
	Number of instances analysis		excluding patients with outcome prior to the time window<br>excluding patients with zero outcomes<br>counts are grouped by date
	Risk analysis		excluding patients with outcome prior to the time window
**GI Bleed**			
**Outcome definition**			
	Diagnosis	UMLS:ICD10CM:K92.2	Gastrointestinal hemorrhage, unspecified
	Diagnosis	UMLS:ICD10CM:K25.0	Acute gastric ulcer with hemorrhage
	Diagnosis	UMLS:ICD10CM:K25.4	Chronic or unspecified gastric ulcer with hemorrhage
	Diagnosis	UMLS:ICD10CM:K26.4	Chronic or unspecified duodenal ulcer with hemorrhage
	Diagnosis	UMLS:ICD10CM:K26.0	Acute duodenal ulcer with hemorrhage
**Settings for the performed analyses**			
	Kaplan - Meier survival analysis		excluding patients with outcome prior to the time window

Number of instances analysis	excluding patients with outcome prior to the time window<br>excluding patients with zero outcomes<br>counts are grouped by date
Risk analysis	excluding patients with outcome prior to the time window

Supplementary Table S4. ICD Codes

Variable	ICD 10 Code
**Base Population**	
Pulmonary embolism	UMLS:ICD10CM:I26
Acute embolism and thrombosis of veins of upper extremity	UMLS:ICD10CM:I82.6
Acute embolism and thrombosis of deep veins of lower extremity	UMLS:ICD10CM:I82.4
Mode of Admission	UMLS:HL7V3.0:VisitType:EMER
	UMLS:HL7V3.0:VisitType:ACUTE
	UMLS:HL7V3.0:VisitType:NONAC
	UMLS:HL7V3.0:VisitType:IMP
**Medication**	
Apixaban	NLM:RXNORM:1364430
Rivaroxaban	NLM:RXNORM:1114195
**Outcomes**	
All-Cause Mortaity	Deceased
Major Bleeding (Composite)	UMLS:ICD10CM:I60
	UMLS:ICD10CM:I61
	UMLS:ICD10CM:I62
	UMLS:ICD10CM:K25.0
	UMLS:ICD10CM:K25.4
	UMLS:ICD10CM:K26.4
	UMLS:ICD10CM:K26.0
	UMLS:ICD10CM:M25.0
	UMLS:ICD10CM:H35.6
	UMLS:ICD10CM:K92.2
	UMLS:ICD10CM:D62
	UMLS:CPT:36430
	UMLS:ICD10CM:R58

	UMLS:ICD10CM:R31
	UMLS:ICD10CM:N93.9
Recurrent VTE	UMLS:ICD10CM:I26
	UMLS:ICD10CM:I82.6
	UMLS:ICD10CM:I82.4
Non-Major Bleed	UMLS:ICD10CM:R58
	UMLS:ICD10CM:R31
	UMLS:ICD10CM:N93.9
Major Bleeding	UMLS:ICD10CM:I61
	UMLS:ICD10CM:I62
	UMLS:ICD10CM:I60
	UMLS:ICD10CM:K25.0
	UMLS:ICD10CM:K25.4
	UMLS:ICD10CM:K26.4
	UMLS:ICD10CM:K26.0
	UMLS:ICD10CM:M25.0
	UMLS:ICD10CM:H35.6
	UMLS:ICD10CM:D62
	UMLS:ICD10CM:K92.2
ICH	UMLS:ICD10CM:I61
	UMLS:ICD10CM:I62
	UMLS:ICD10CM:I60
GI Bleed	UMLS:ICD10CM:K92.2
	UMLS:ICD10CM:K25.0
	UMLS:ICD10CM:K25.4
	UMLS:ICD10CM:K26.4
	UMLS:ICD10CM:K26.0

Supplementary Table S5. Outcomes, Subgrouped by Weight > 120 Kg (Obese)

Outcome	Apixaban and VTE Group (n/N)	Rivaroxaban and VTE Group (n/N)	HR (95% CI)	Log-rank p value
Primary Outcome				
**Recurrent VTE**				
*At 3-Month**	111/484	106/484	1.03 (0.79 - 1.34)	0.837
*At 6-Month**	151/484	135/484	1.12 (0.88 - 1.41)	0.355
*At 1-Year**	166/484	153/484	1.10 (0.88 - 1.37)	0.394
*At 2-Year**	176/484	160/484	1.13 (0.91 - 1.40)	0.263
Secondary Outcome				
**All-Cause Mortality**				
At 6-Month	12/481	15/483	0.80 (0.37 - 1.70)	0.555
At 1-Year	18/481	19/483	0.95 (0.50 - 1.81)	0.871
At 2-Year	18/486	23/486	0.82 (0.45 - 1.53)	0.538
**Major Bleeding (Composite)**				
At 6-Month	13/440	19/440	0.69 (0.34 - 1.39)	0.296
At 1-Year	16/440	26/440	0.62 (0.33 - 1.15)	0.127
At 2-Year	12/443	32/443	0.39 (0.20 - 0.76)	0.004

- *HR = Hazard Ratio; CI = Confidence Interval*
- *n = population with outcome*
- *N = total population of cohort*
- *Denominators vary across outcomes due to outcome-specific cohort definitions, eligibility criteria, and variable data availability within the TriNetX database.*

** Outcome with a lag window of 30 days (from day 1 to day 30)*

Supplementary Table S6. Outcomes, Subgrouped by Cancer

Outcome	Apixaban and VTE Group (n/N)	Rivaroxaban and VTE Group (n/N)	HR (95% CI)	Log-rank p value
Primary Outcome				
**Recurrent VTE**				
*At 3-Month**	236/1,067	257/1,067	0.95 (0.80 - 1.13)	0.57
*At 6-Month**	311/1,067	317/1,067	1.02 (0.87 - 1.19)	0.832
*At 1-Year**	335/1,067	347/1,067	1.01 (0.87 - 1.17)	0.897
*At 2-Year**	350/1,067	367/1,067	1.00 (0.87 - 1.16)	0.959
Secondary Outcome				
**All-Cause Mortality**				
At 3-Month	186/1,058	176/1,059	1.08 (0.88 - 1.32)	0.483
At 6-Month	228/1,058	231/1,059	1.01 (0.84 - 1.22)	0.889
At 1-Year	271/1,058	277/1,059	1.02 (0.86 - 1.21)	0.816
At 2-Year	308/1,058	320/1,059	1.02 (0.88 - 1.20)	0.768
**Major Bleeding (Composite)**				
At 3-Month	98/855	78/878	1.32 (0.98 - 1.78)	0.065
At 6-Month	117/855	95/878	1.30 (1.00 - 1.71)	0.054
At 1-Year	129/855	105/878	1.31 (1.02 - 1.70)	0.037
At 2-Year	146/855	121/878	1.32 (1.04 - 1.68)	0.023
**Non-Major Bleed**				
At 3-Month	36/984	28/992	1.32 (0.81 - 2.17)	0.264
At 6-Month	44/984	34/992	1.34 (0.86 - 2.10)	0.197
At 1-Year	50/984	36/992	1.45 (0.95 - 2.23)	0.085
At 2-Year	57/984	46/992	1.34 (0.91 - 1.97)	0.142
**Major Bleeding**				
At 3-Month	44/948	29/952	1.54 (0.96 - 2.46)	0.068
At 6-Month	54/948	37/952	1.49 (0.98 - 2.27)	0.059
At 1-Year	63/948	43/952	1.52 (1.03 - 2.24)	0.033
At 2-Year	70/948	53/952	1.40 (0.98 - 2.01)	0.062
**GI Bleed**				
At 3-Month	13/1,032	15/1,032	0.88 (0.42 - 1.85)	0.733

At 6-Month	17/1,032	18/1,032	0.97 (0.50 - 1.87)	0.915
At 1-Year	21/1,032	22/1,032	0.99 (0.55 - 1.80)	0.975
At 2-Year	24/1,032	27/1,032	0.95 (0.55 - 1.64)	0.844

- *HR = Hazard Ratio; CI = Confidence Interval*
- *n = population with outcome*
- *N = total population of cohort*
- *Denominators vary across outcomes due to outcome-specific cohort definitions, eligibility criteria, and variable data availability within the TriNetX database.*

** Outcome with a lag window of 30 days (from day 1 to day 30)*

Supplementary Table S7. Sensitivity Analyses for Recurrent VTE

Outcome	Apixaban and VTE Group (n/N)	Rivaroxaban and VTE Group (n/N)	HR (95% CI)	Log-rank p value
Primary Outcome				
**Recurrent VTE**				
At 3-Month (without lag Window)	4,091/8,247	4,090/8,247	1.01 (0.97 - 1.05)	0.682
At 30 days (day1 to day 30)	3,532/8,247	3,571/8,247	0.99 (0.95 - 1.04)	0.771

- *HR = Hazard Ratio; CI = Confidence Interval*
- *n = population with outcome*
- *N = total population of cohort*
- *Denominators vary across outcomes due to outcome-specific cohort definitions, eligibility criteria, and variable data availability within the TriNetX database.*

Supplementary Figure S1. Propensity Score Matching

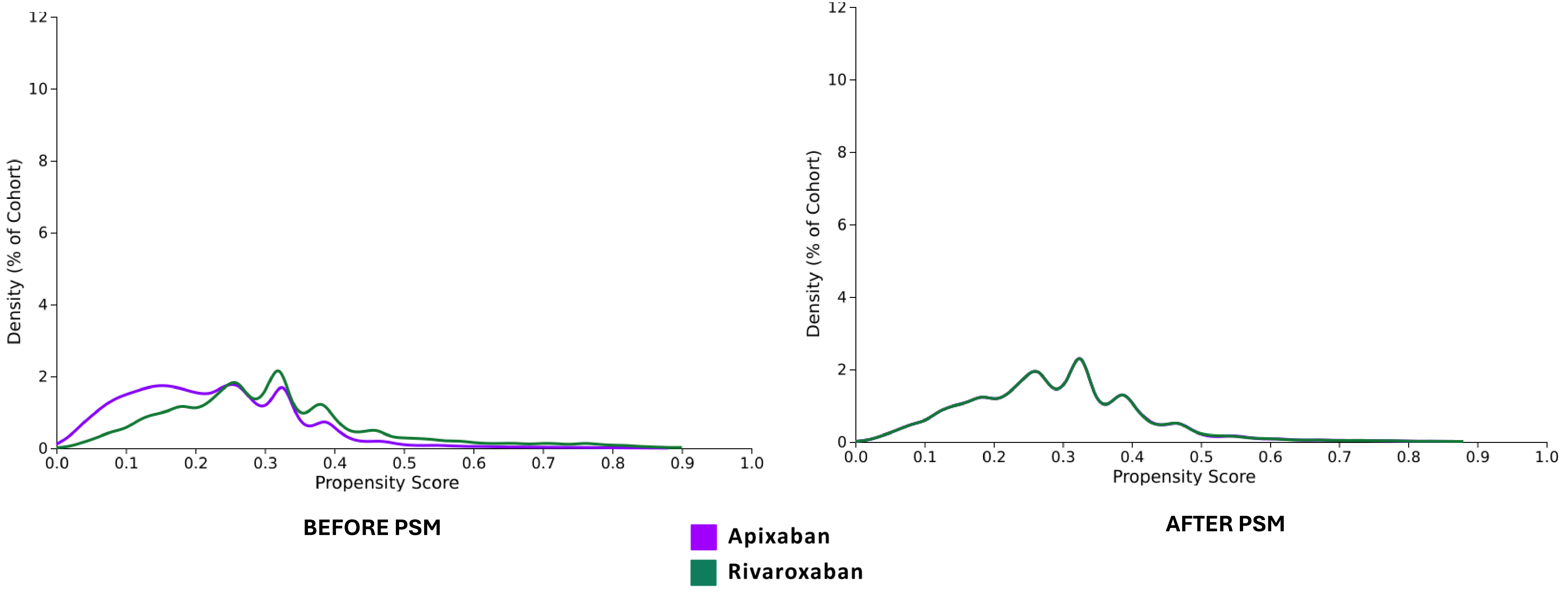

Supplementary Figure S2. Recurrent VTE, Subgrouped by Weight > 120 Kg (Obese) at 1-Year

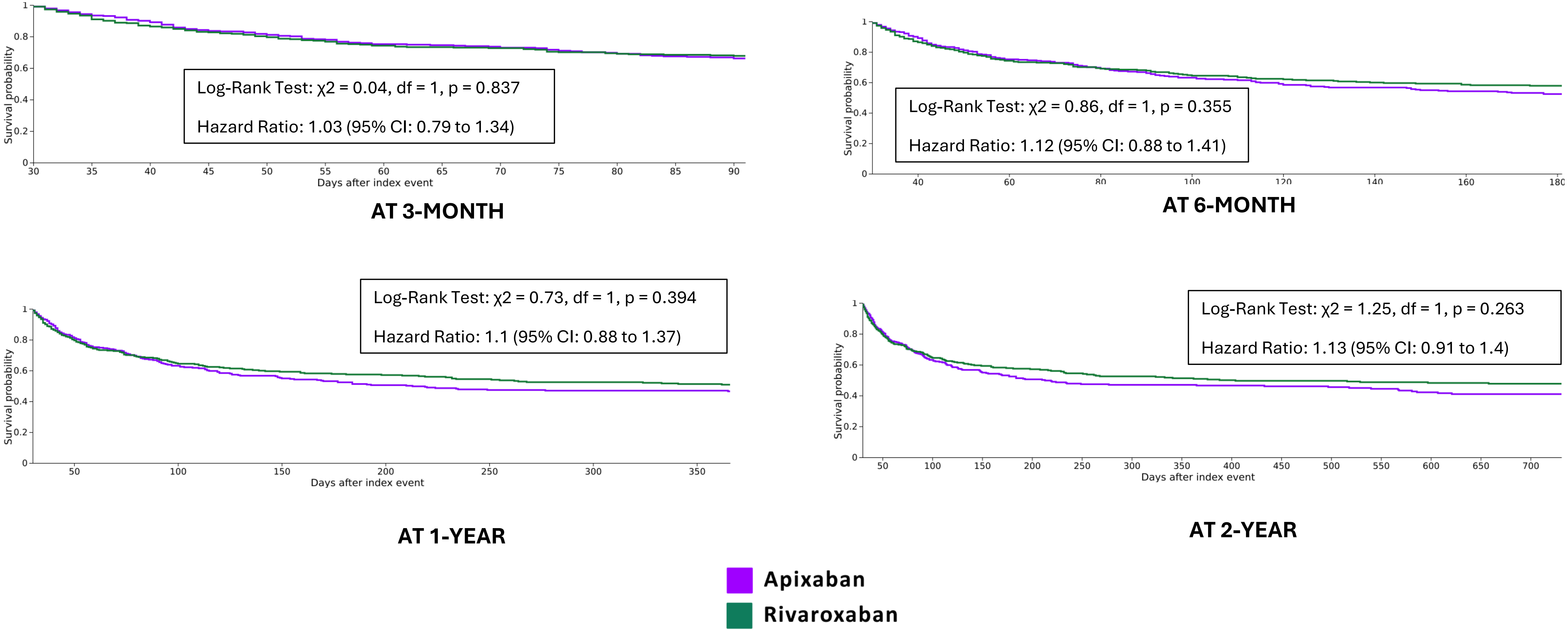

Supplementary Figure S3. Recurrent VTE, Subgrouped by Cancer

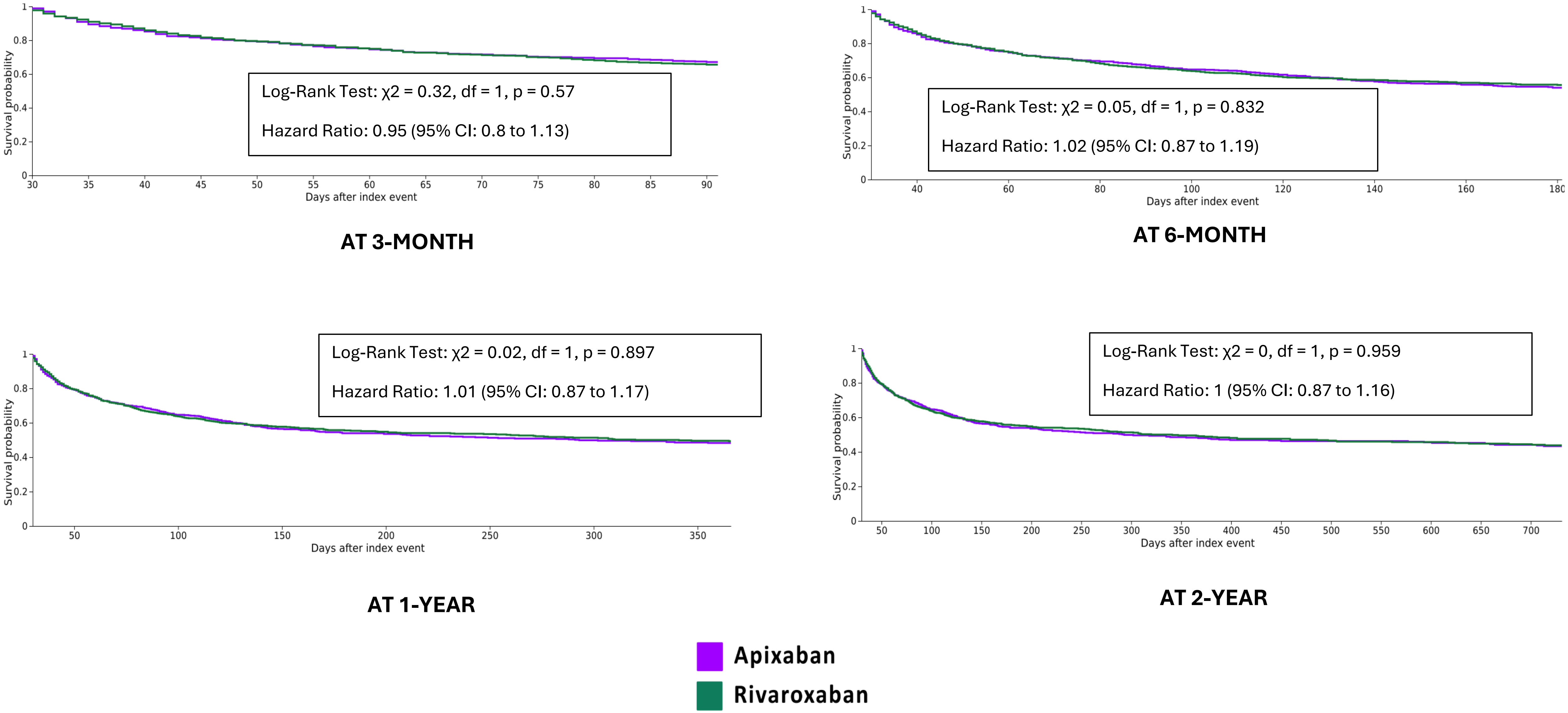

Supplementary Figure S4. All-cause Mortality

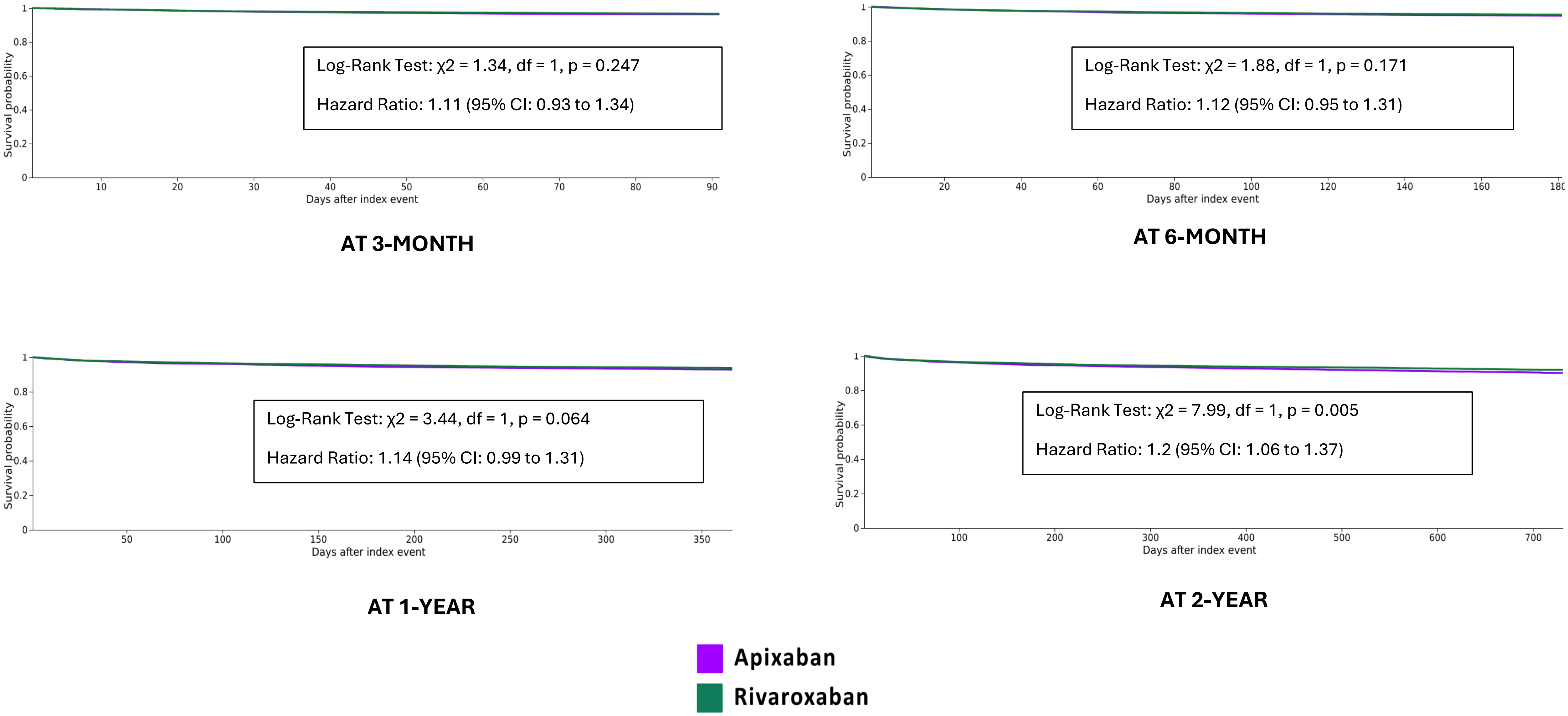

Supplementary Figure S5. All-cause Mortality, Subgrouped by Weight > 120 Kg (Obese)

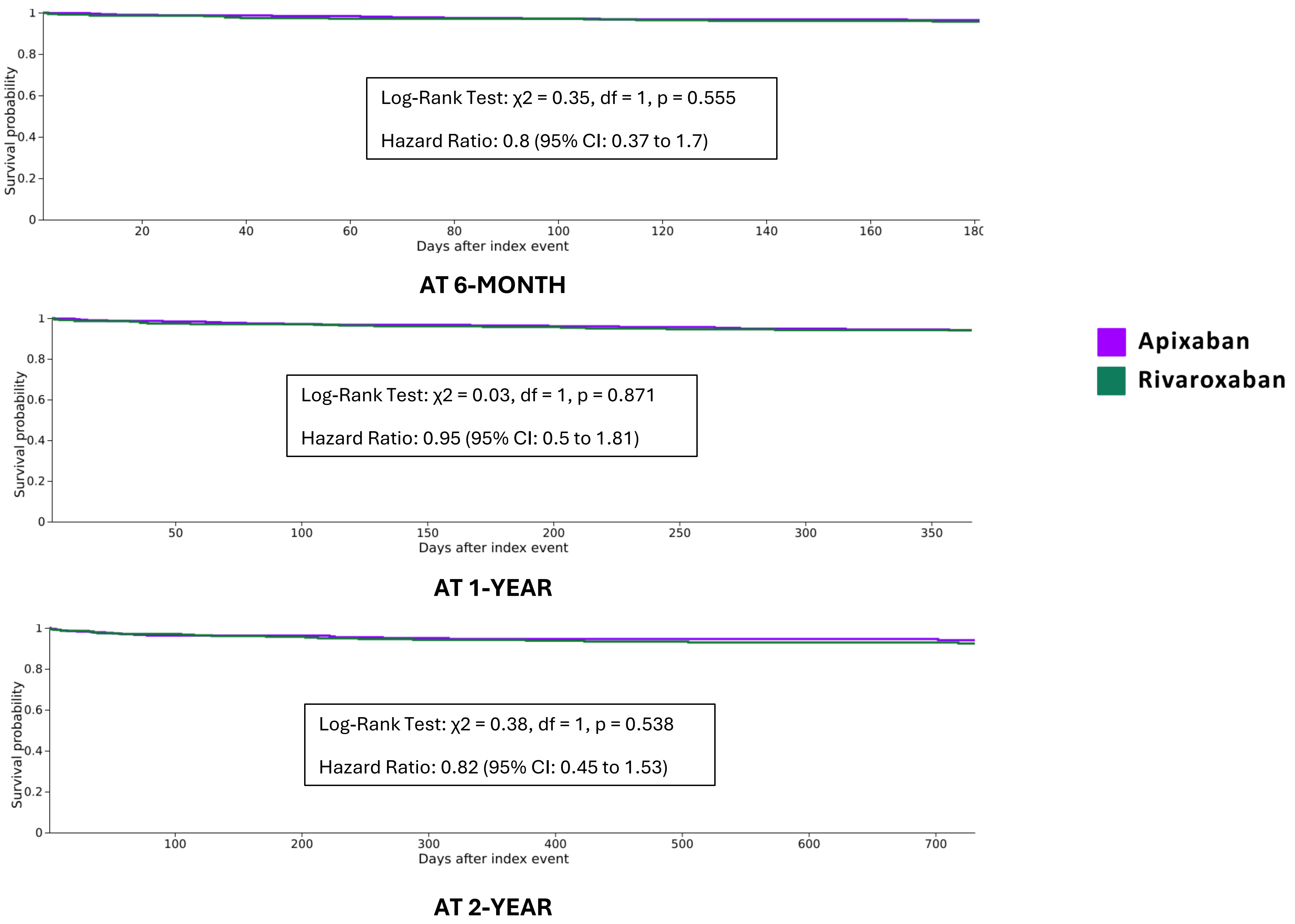

Supplementary Figure S6. All-cause Mortality, Subgrouped by Cancer

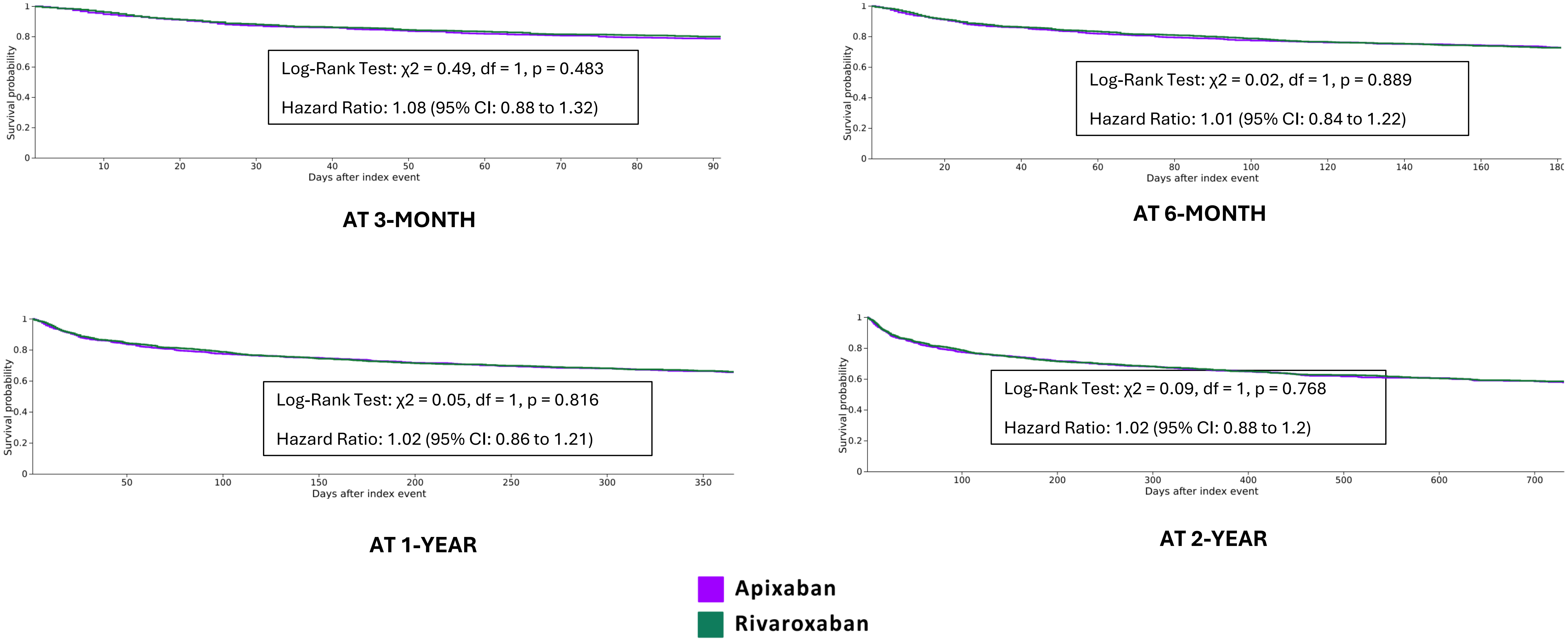

Supplementary Figure S7. Major Bleeding (Composite)

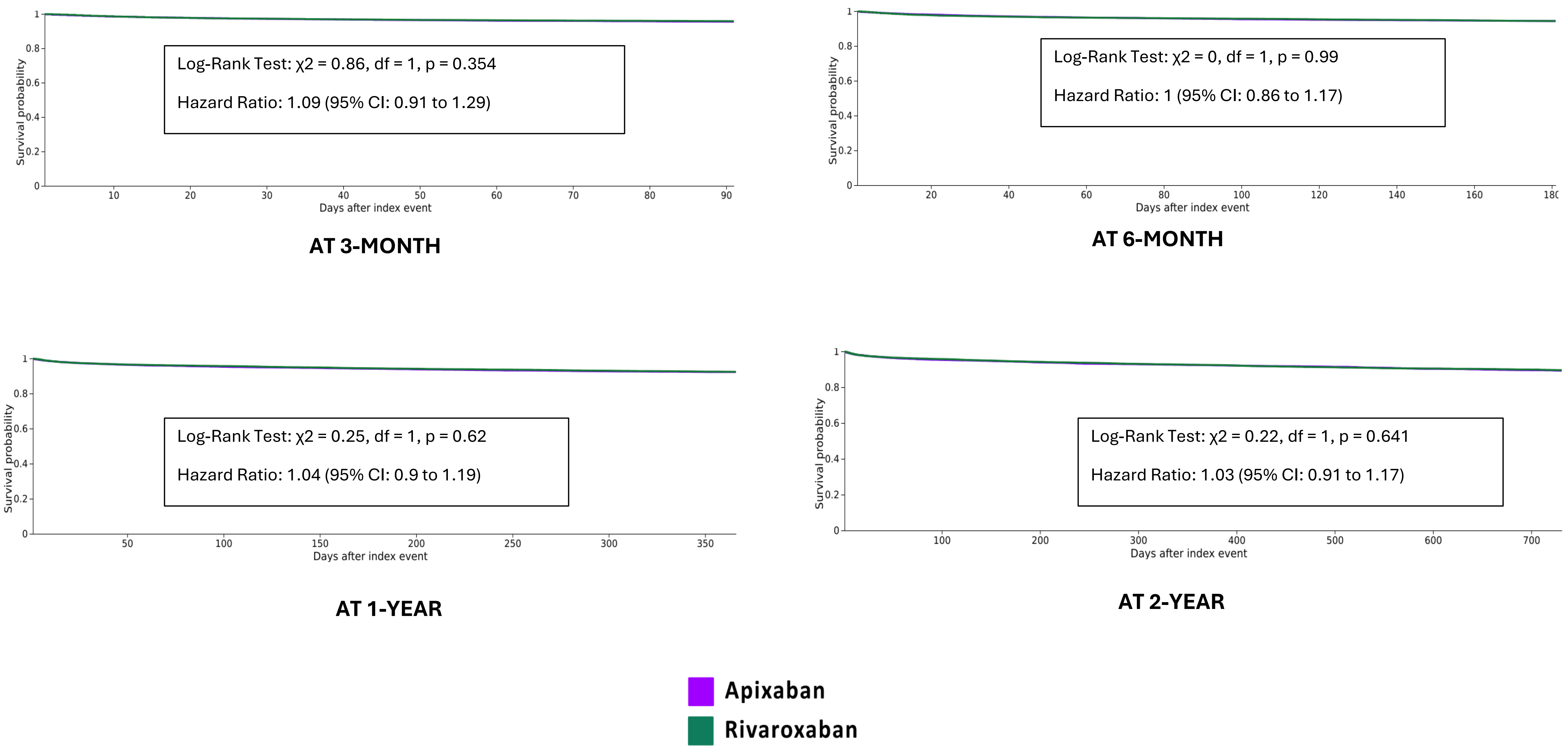

Supplementary Figure S8. Major Bleeding (Composite), Subgrouped by Weight > 120 Kg (Obese)

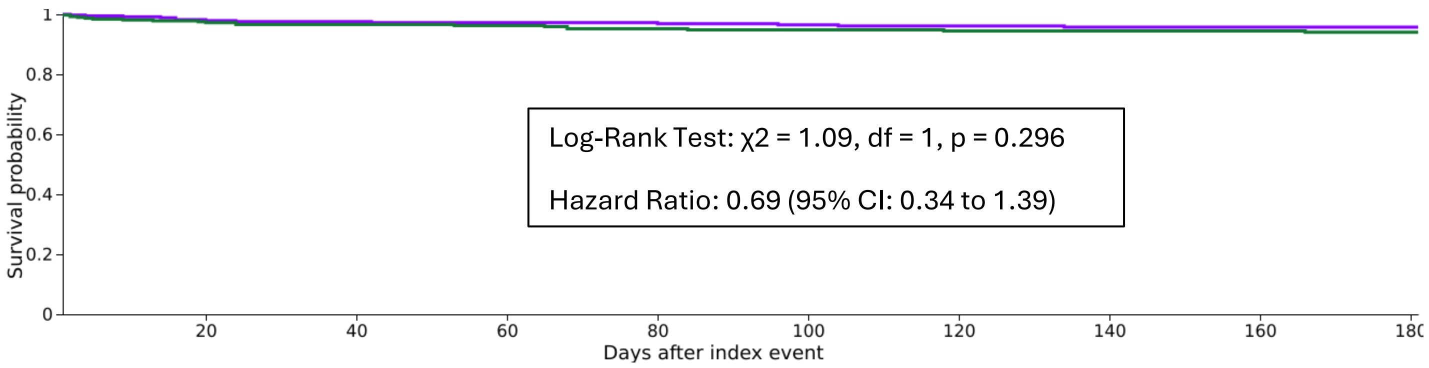

AT 6-MONTH

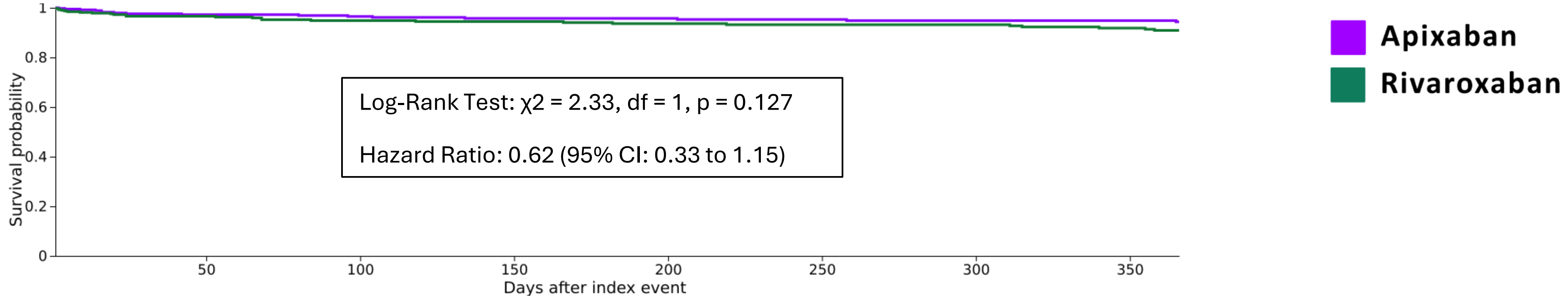

AT 1-YEAR

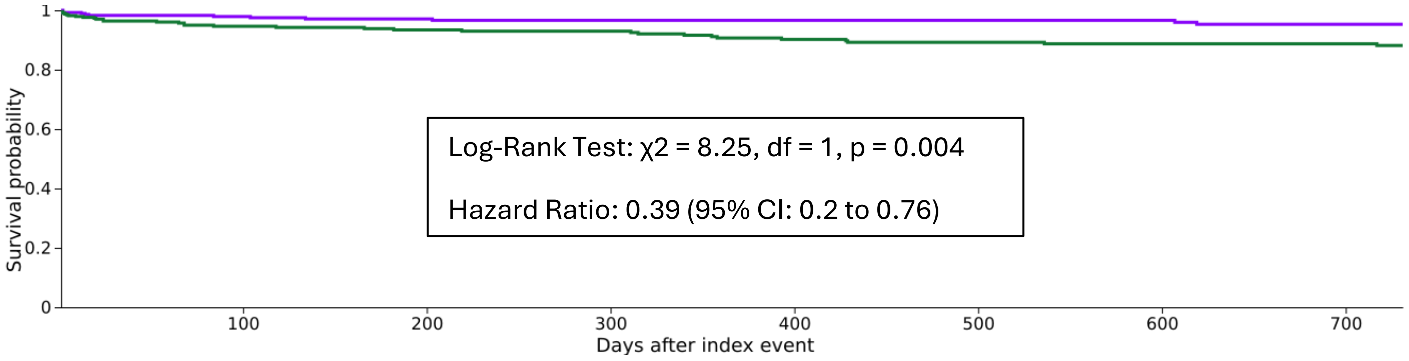

AT 2-YEAR

Supplementary Figure S9. Major Bleeding (Composite), Subgrouped by Cancer

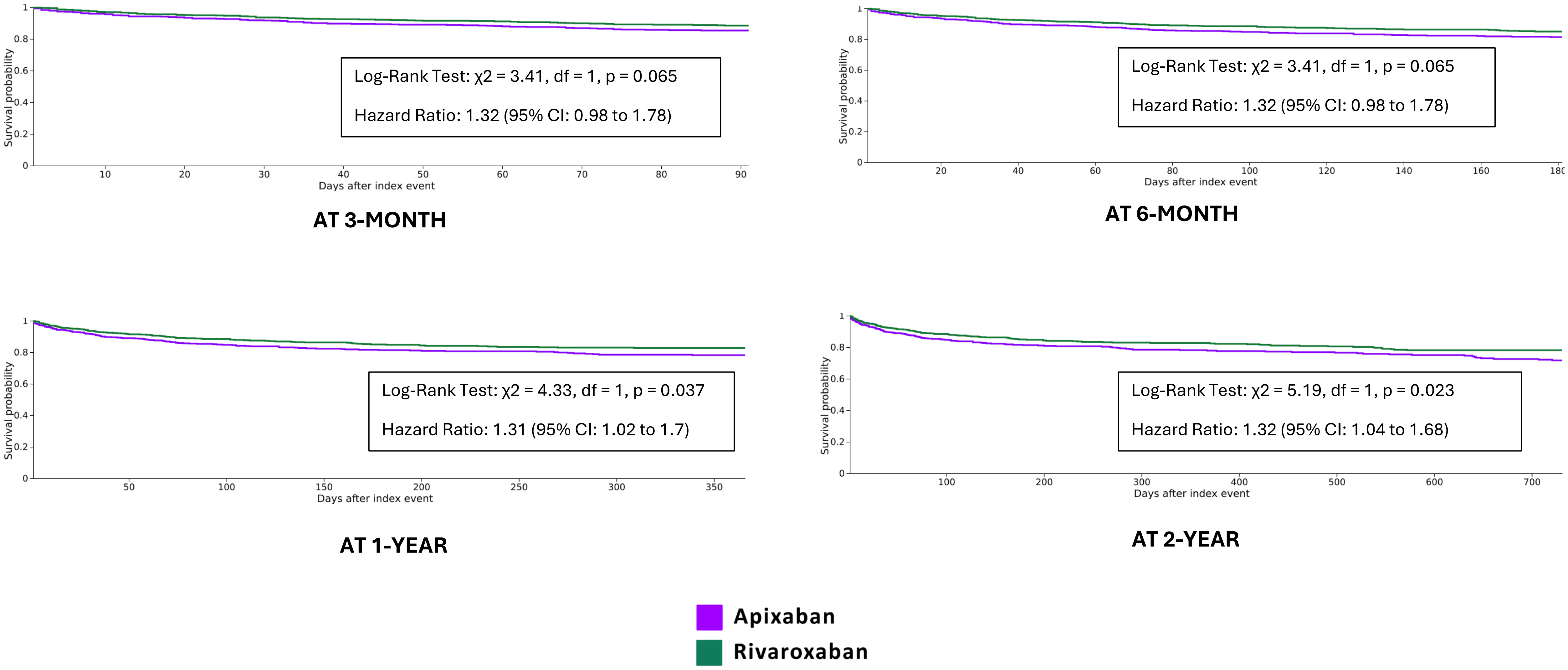

Supplementary Figure S10. Non-Major Bleed

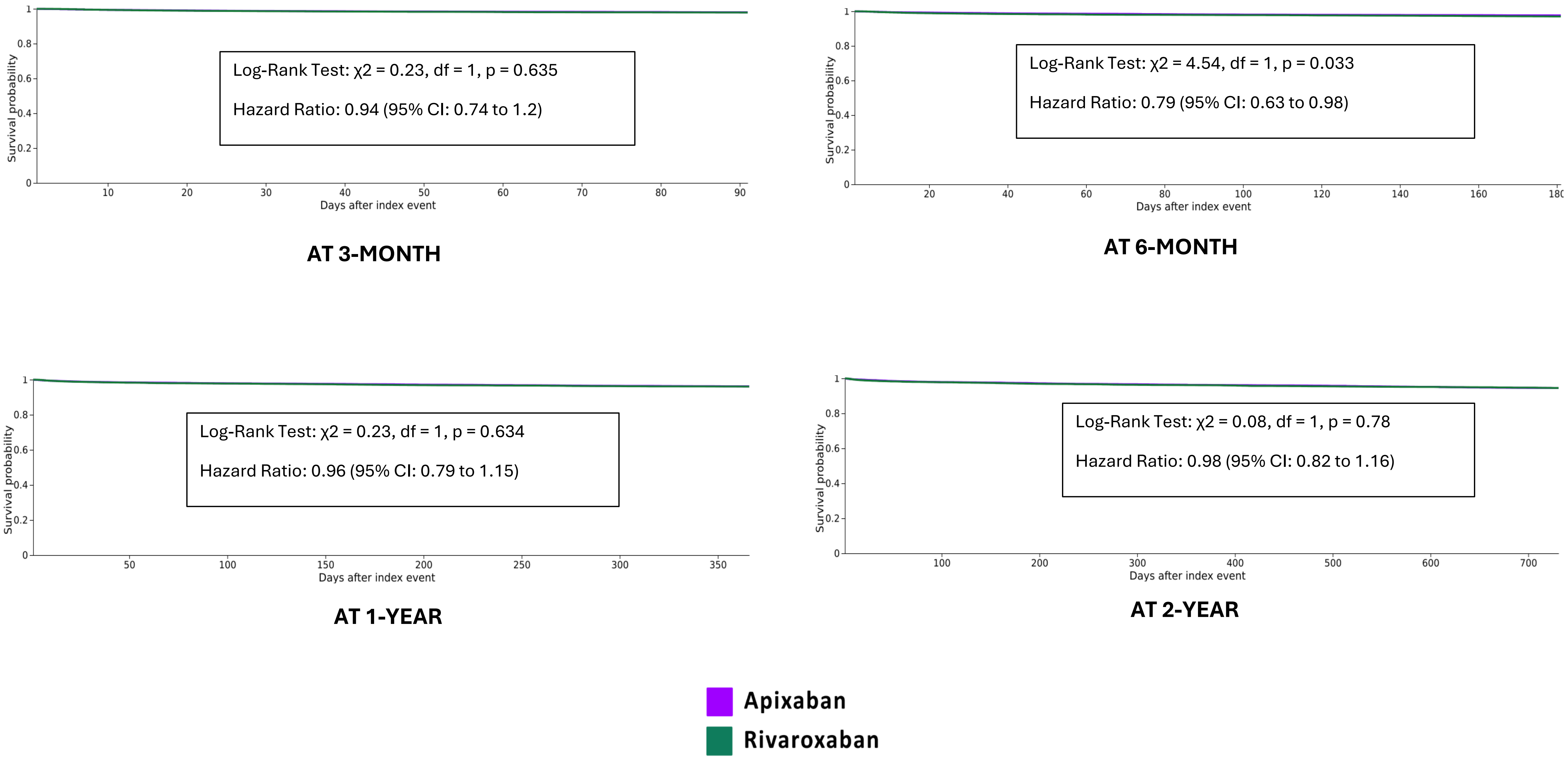

Supplementary Figure S11. Non-Major Bleed, Subgrouped by Cancer

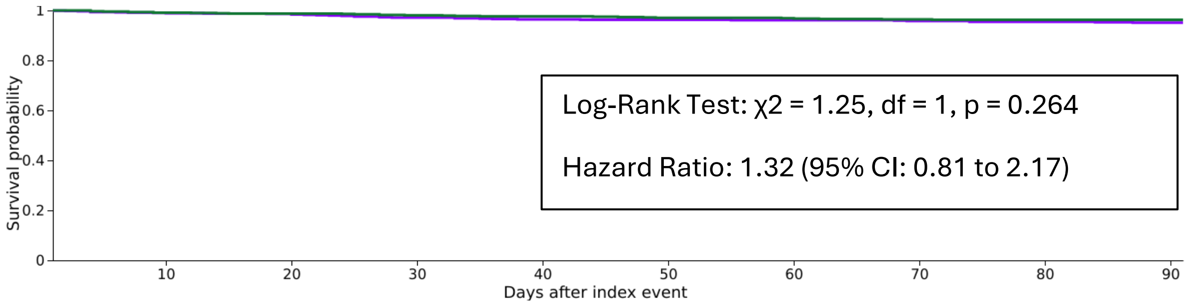

AT 3-MONTH

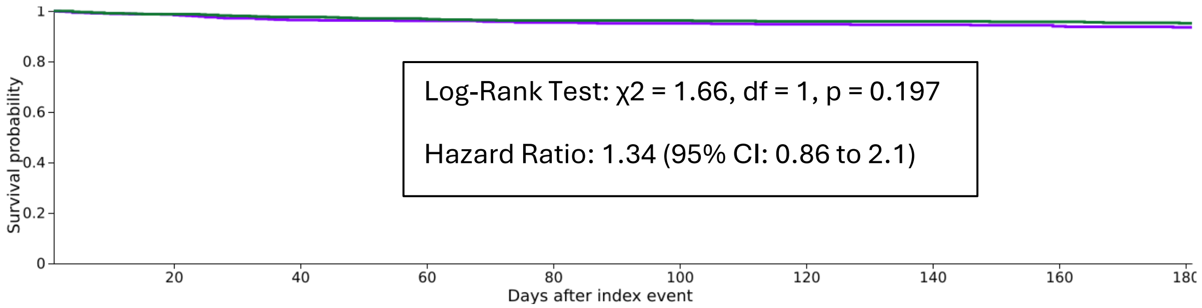

AT 6-MONTH

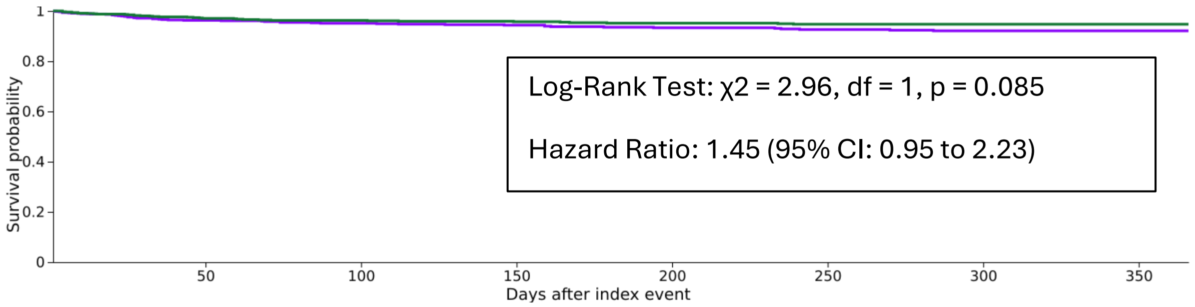

AT 1-YEAR

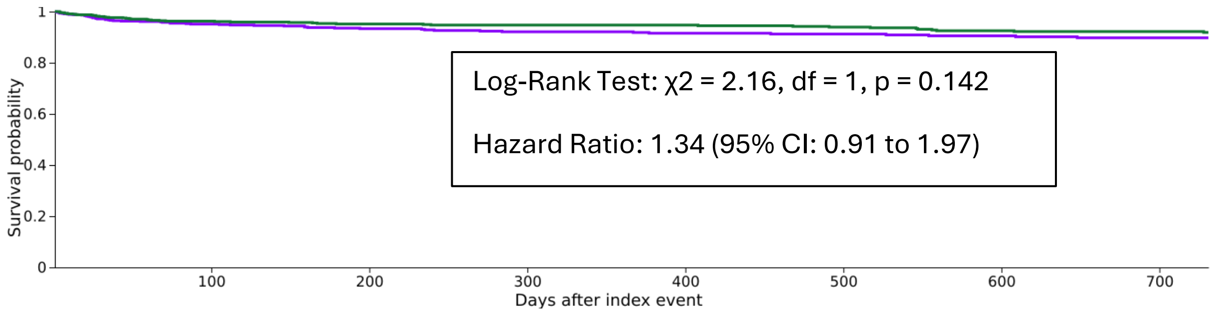

AT 2-YEAR

Apixaban
Rivaroxaban

Supplementary Figure S12. Major Bleeding

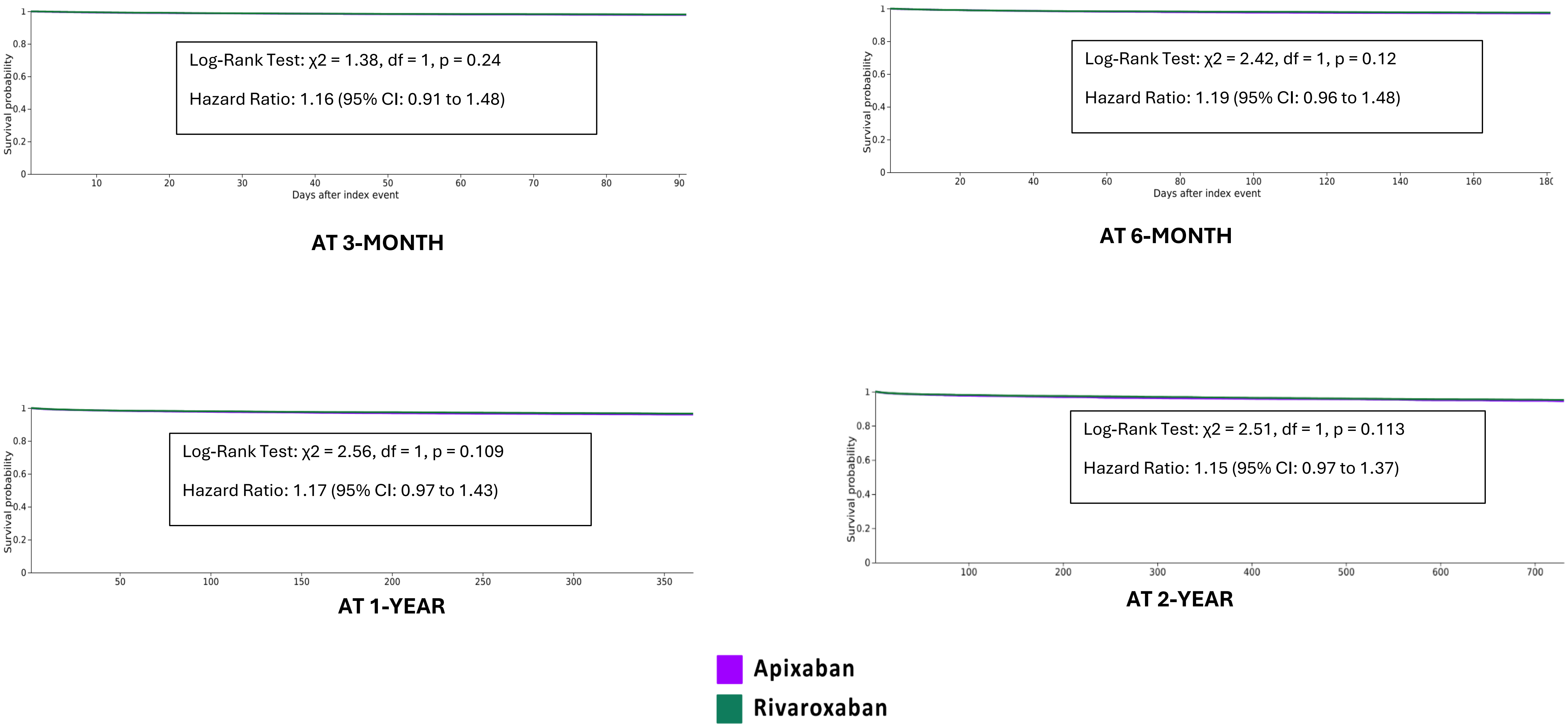

Supplementary Figure S13. Major Bleeding, Subgrouped by Cancer

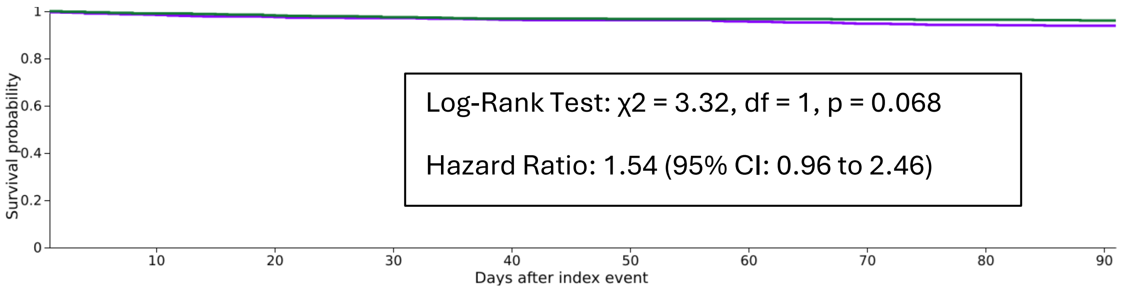

AT 3-MONTH

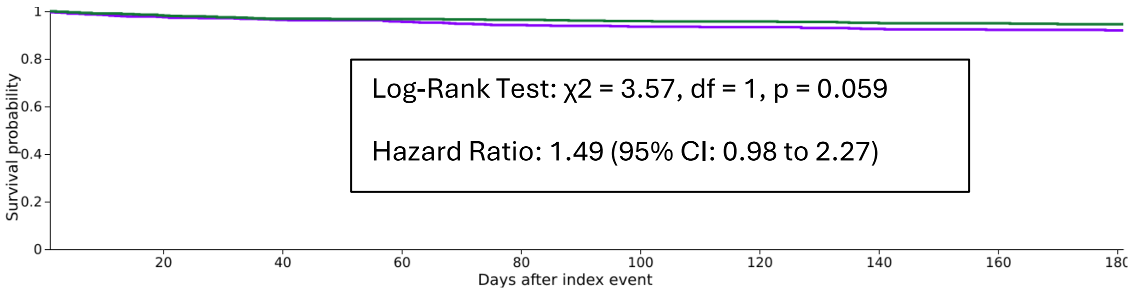

AT 6-MONTH

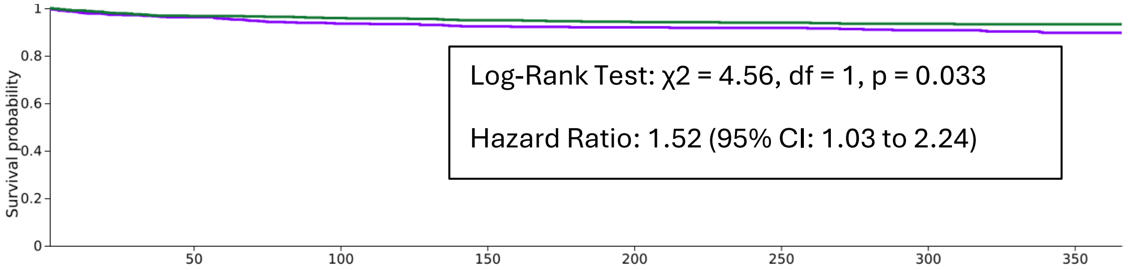

AT 1-YEAR

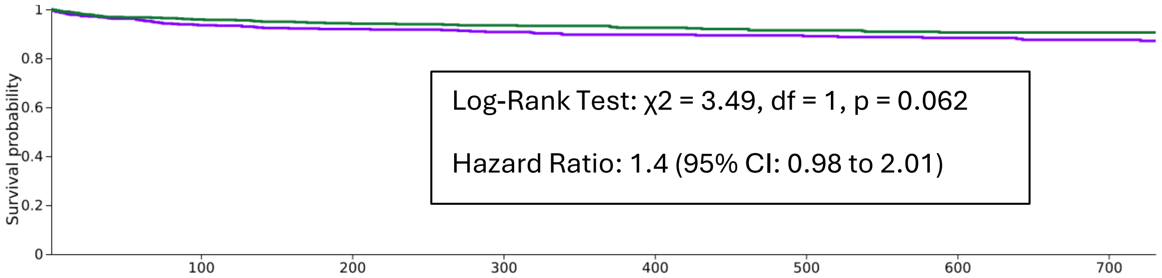

AT 2-YEAR

Apixaban
Rivaroxaban

Supplementary Figure S14. ICH

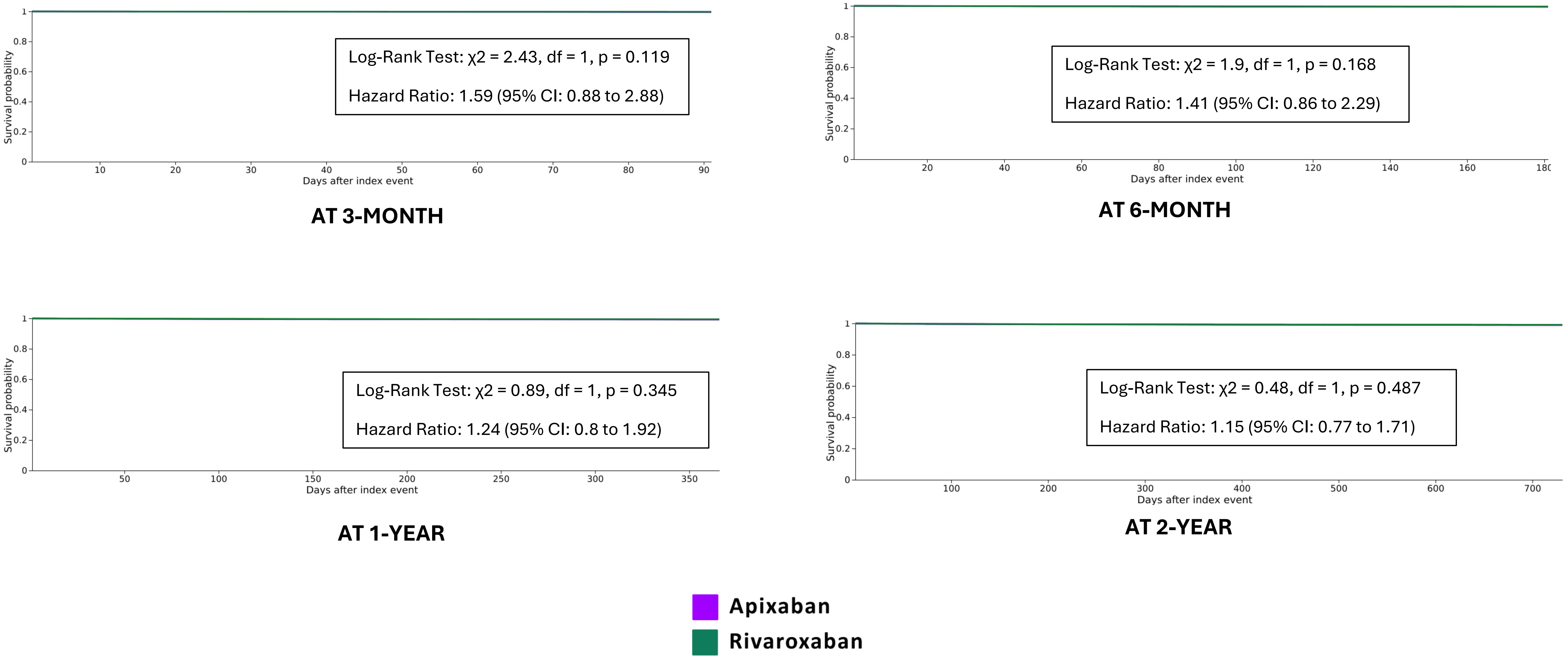

Supplementary Figure S15. GI Bleed

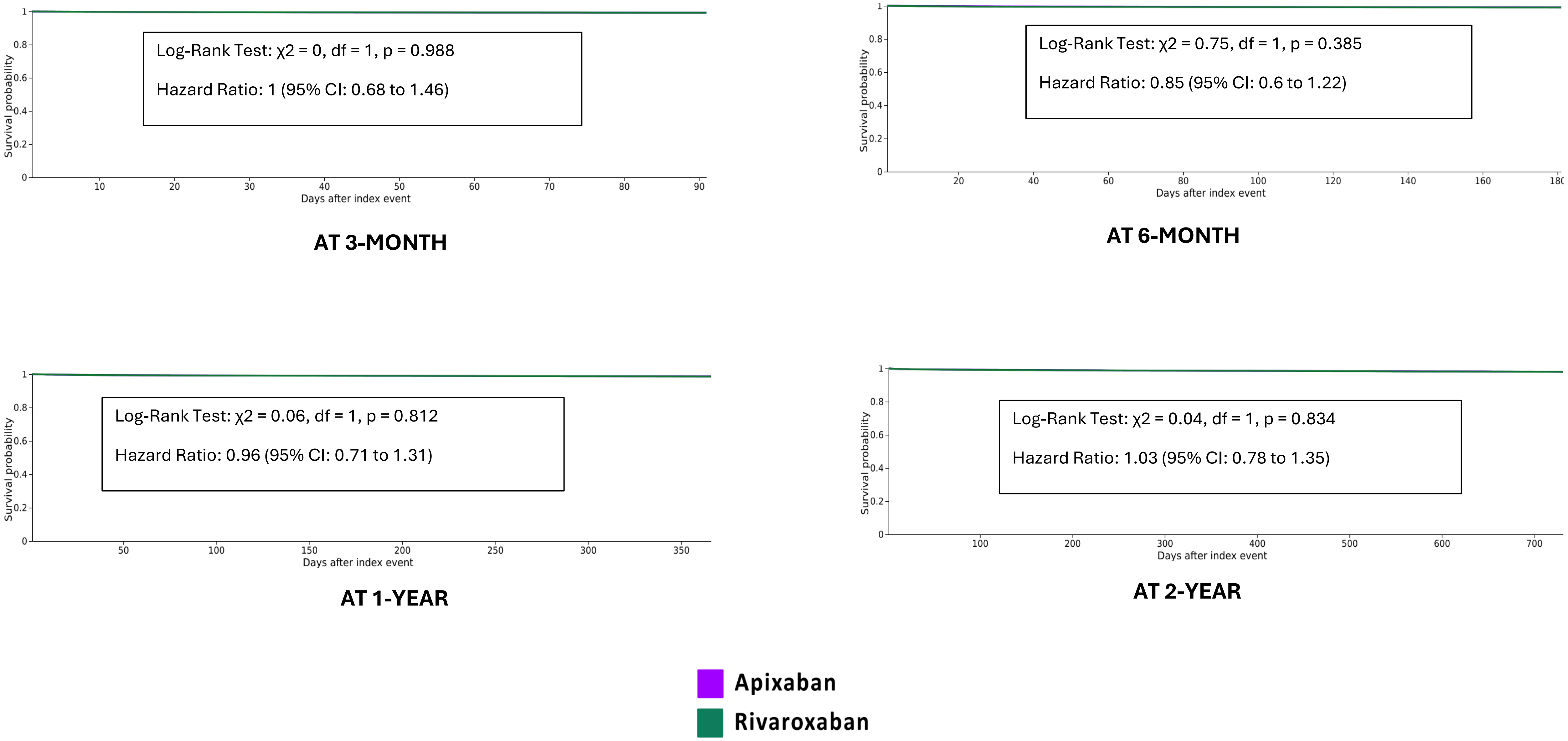

Supplementary Figure S16. GI Bleed, Subgrouped by Cancer

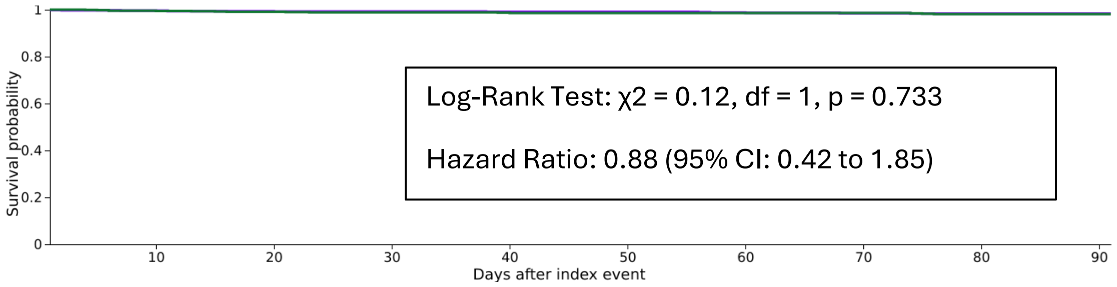

AT 3-MONTH

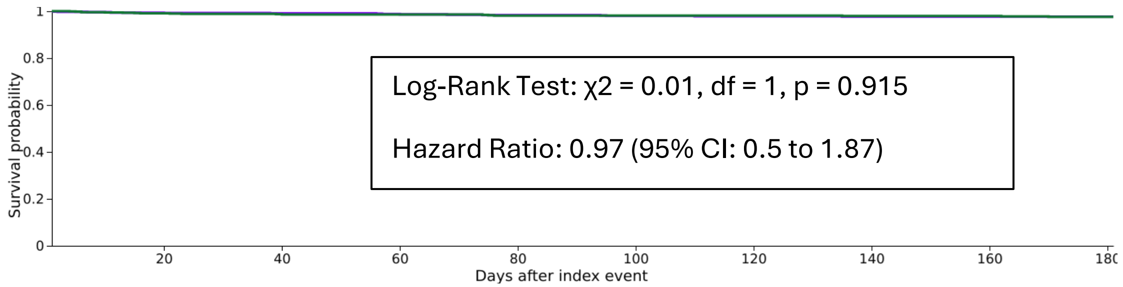

AT 6-MONTH

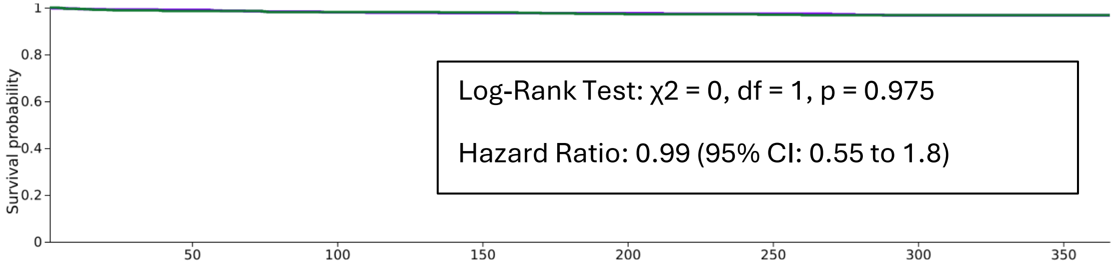

AT 1-YEAR

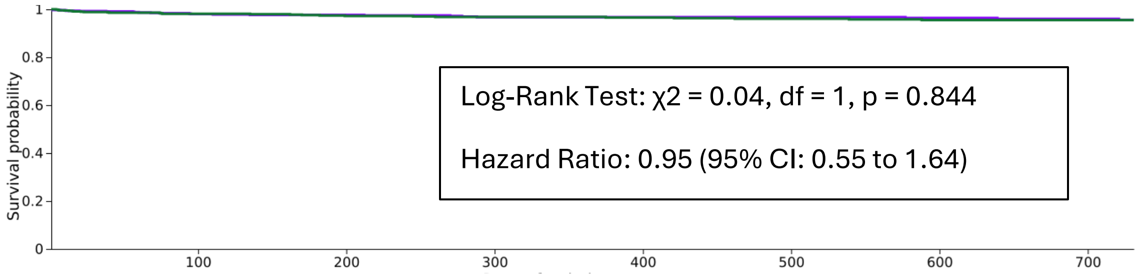

AT 2-YEAR

Apixaban
Rivaroxaban
